# Supplementary material for: Menstrual health intervention and school attendance in Uganda (MENISCUS-2): a pilot intervention study
Source: BMJ Open. 2020 Feb 4;10(2):e031182. doi: 10.1136/bmjopen-2019-031182 (PMC7044877; doi:10.1136/bmjopen-2019-031182)
Supplement: Supplementary data [file bmjopen-2019-031182supp001.pdf]

Supplementary Table 1: Description of the MENISCUS intervention

**MENISUS intervention**<br>**1. Puberty education**<br>**2. Drama skit**<br>**3. Menstrual management kit & training**<br>**4. Pain management**<br>**5. Water, Sanitation and Hygiene (WASH) improvements**
**Materials & procedures**
1. Secondary year 2 teachers from both intervention schools and other relevant teachers selected by school management received 2 days of training in how to deliver puberty education according to government guidelines using the draft National Training of Trainers Manual on MHM compiled by the Ugandan Ministry of Education and Sports (MoES).<br>2. School Drama Groups with students from Secondary year 1 to year 4 received two facilitated drama skit sessions (one on menstrual health and one on the drama skit process) followed by follow-up visits to drama group practices. The MHM drama was performed by the drama groups at a parental meeting at each school (an annual general meeting and an specifically organised parental meeting). The meetings were attended by teachers, parents of secondary school students and some secondary year 2 students.<br>3. Secondary year 2 girl and boy students were invited to participate in an MHM training session. Secondary year 2 girl students were provided with a menstrual kit consisting of a pack of AFRIpads reusable pads, a towel, soap, water bottle, knickers and menstrual calendar and an educational session on safe use and care of reusable menstrual products as well as pain management methods. Follow-up sessions were provided throughout the school year.<br>4. Students were provided with one voucher at baseline and throughout the study to redeem painkillers at school or a local pharmacy. All used vouchers were replaced per month.<br>5. WASH improvements consisted of installing locks, repairing broken doors, providing bins and toilet paper holders fixed to the wall, liquid hand washing soap and water drums.
**Who provided**
1. An independent educational consultant with support from WoMena Uganda staff members trained teachers from each school who are responsible for delivering puberty education in 2-days of puberty education training. The educational consultant was a professional trainer with expertise in education management.WoMena Uganda is a non-governmental organisation with expertise in programme design, monitoring and evaluation and education for menstrual health interventions. WoMena has a training team of young Ugandan menstrual health trainers (aged 20 -28, with educational backgrounds in social care, nursing, education), led by the Training Coordinator. The puberty education sessions were supported by the Training Coordinator and a trainer.<br>2. Two facilitated drama skit sessions were delivered by the WoMena Uganda Training Coordinator (supported by a Drama Skit facilitation guide developed by WoMena Uganda). Follow-up sessions with the Drama Groups were led by the school Drama Teacher and supported by an independent drama skit consultant, who was engaged in Drama Skit activities during MENISCUS-1 (a previous formative study carried out in Entebbe).<br>3. The MHM training session was delivered by selected schoolteachers and peers who had been trained in its delivery by WoMena Uganda. Training sessions were supported by a

team of six Womena Uganda trainers and the Training Coordinator. Follow-up sessions were provided by WoMena Uganda.<br>4. Painkiller vouchers could be redeemed for painkillers (paracetamol) from selected school teachers, nurse, senior women teachers and a local pharmacy.<br>5. WASH improvements were made by MRC/UVRI and maintained by the schools.
**How**
1. An education consultant and WoMena Uganda provided 2 days of group training in puberty education to teachers.<br>2. Two facilitated Drama Skit introduction sessions were held in each school by WoMena Uganda. Follow up sessions throughout terms 3 and 1 (of year 3) were carried out by the drama skit consultant. The drama performances were arranged by the school Drama Teachers in collaboration with school management as part of parental meetings.<br>3. Menstrual management kits and training of school teachers and peers was provided in group training sessions by WoMena Uganda<br>4. Painkillers and vouchers were delivered to schools / students by the MRC/UVRI and LSHTM research team..<br>5. WASH improvements were made by MRC/UVRI and LSHTM and maintained by the schools.
**Where**
Staff training in puberty education was conducted outside the schools. All other activities with students and staff were conducted in schools.
**When and how much**
1. Two days of training in puberty education to teachers delivered in April 2017.<br>2. Two, two-hour introductions facilitated by WoMena Uganda were delivered in October and November 2017. 21 follow up visits to drama skit practices were carried out between November 2017 and June 2018 (10 follow-up session planned). Two drama skit performances were carried out in July 2018.<br>3. Two, one-day Training of Trainers sessions delivered by WoMena Uganda in two schools to 11 female teachers, 11 female students and 2 males (drama teacher and school nurse) in May 2017. A one-day refresher training was held in September 2017, for selected female students (5) and female teachers (6) before delivery of training to students (not planned). Training to Secondary year 2 students (boys and girls) was delivered over 8 days in October and November (planned 7 days) over 17 training sessions (15 planned).
