# Supplementary material for: Menstrual health intervention and school attendance in Uganda (MENISCUS-2): a pilot intervention study
Source: BMJ Open. 2020 Feb 4;10(2):e031182. doi: 10.1136/bmjopen-2019-031182 (PMC7044877; doi:10.1136/bmjopen-2019-031182)
Supplement: Author's manuscript [file bmjopen-2019-031182.draft_revisions.pdf]

BMJ Open

BMJ Open is committed to open peer review. As part of this commitment we make the peer review history of every article we publish publicly available.

When an article is published we post the peer reviewers' comments and the authors' responses online. We also post the versions of the paper that were used during peer review. These are the versions that the peer review comments apply to.

The versions of the paper that follow are the versions that were submitted during the peer review process. They are not the versions of record or the final published versions. They should not be cited or distributed as the published version of this manuscript.

BMJ Open is an open access journal and the full, final, typeset and author-corrected version of record of the manuscript is available on our site with no access controls, subscription charges or pay-per-view fees (http://bmjopen.bmj.com).

If you have any questions on BMJ Open's open peer review process please email info.bmjopen@bmj.com

BMJ Open

Menstrual health intervention and school attendance in Uganda (MENISCUS-2): A pilot intervention study

Journal:	*BMJ Open*
Manuscript ID	bmjopen-2019-031182
Article Type:	Original research
Date Submitted by the Author:	20-Apr-2019

Complete List of Authors:	Kansiime, Catherine; Medical Research Council / Uganda Virus Research Institute & London School of Hygiene & Tropical Medicine Uganda Research Unit<br>Hytti, Laura; WoMena Uganda<br>Nalugya, Ruth; Medical Research Council / Uganda Virus Research Institute & London School of Hygiene & Tropical Medicine Uganda Research Unit<br>Nakuya, Kevin; Medical Research Council / Uganda Virus Research Institute & London School of Hygiene & Tropical Medicine Uganda Research Unit<br>Namirembe, Prossy; Medical Research Council / Uganda Virus Research Institute & London School of Hygiene & Tropical Medicine Uganda Research Unit<br>Nakalema, Shamirah; WoMena Uganda<br>Neema, Stella; Makerere University, College of Humanities and Social Science<br>Tanton, Clare; London School of Hygiene and Tropical Medicine, Epidemiology and Population Health<br>Alezuyo, Connie; Palm Tree Academy Uganda<br>Namuli Musoke, Saidat; Uganda Virus Research Institute<br>Torondel, Belen; London School of Hygiene and Tropical Medicine, Faculty of Infectious and Tropical Diseases<br>Francis, Suzanna ; London School of Hygiene and Tropical Medicine, MRC Tropical Epidemiology Group, Faculty of Epidemiology and Population Health<br>Ross, David; World Health Organization, Department of Maternal, Newborn, Child and Adolescent Health<br>Bonell, Christopher; London School of Hygiene and Tropical Medicine, Public Health and Policy<br>Seeley, Janet; London School of Hygiene and Tropical Medicine Faculty of Public Health and Policy; Medical Research Council / Uganda Virus Research Institute & London School of Hygiene & Tropical Medicine Uganda Research Unit<br>Weiss, Helen; London School of Hygiene and Tropical Medicine, MRC Tropical Epidemiology Group, Faculty of Epidemiology and Population Health

Keywords:	Menstrual Health, School attendance, Menstrual Hygiene, Adolescent Health, Education

SCHOLARONE™
Manuscripts

I, the Submitting Author has the right to grant and does grant on behalf of all authors of the Work (as defined in the below author licence), an exclusive licence and/or a non-exclusive licence for contributions from authors who are: i) UK Crown employees; ii) where BMJ has agreed a CC-BY licence shall apply, and/or iii) in accordance with the terms applicable for US Federal Government officers or employees acting as part of their official duties; on a worldwide, perpetual, irrevocable, royalty-free basis to BMJ Publishing Group Ltd ("BMJ") its licensees and where the relevant Journal is co-owned by BMJ to the co-owners of the Journal, to publish the Work in this journal and any other BMJ products and to exploit all rights, as set out in our licence.

The Submitting Author accepts and understands that any supply made under these terms is made by BMJ to the Submitting Author unless you are acting as an employee on behalf of your employer or a postgraduate student of an affiliated institution which is paying any applicable article publishing charge ("APC") for Open Access articles. Where the Submitting Author wishes to make the Work available on an Open Access basis (and intends to pay the relevant APC), the terms of reuse of such Open Access shall be governed by a Creative Commons licence – details of these licences and which Creative Commons licence will apply to this Work are set out in our licence referred to above.

Other than as permitted in any relevant BMJ Author's Self Archiving Policies, I confirm this Work has not been accepted for publication elsewhere, is not being considered for publication elsewhere and does not duplicate material already published. I confirm all authors consent to publication of this Work and authorise the granting of this licence.

For peer review only

Menstrual health interventions and school attendance in Uganda (MENISCUS-2): A pilot intervention study

Catherine Kansiime, Ph.D[a], Laura Hytti, MMSc[b], Ruth Nalugya[a], Kevin Nakuya[a], Prossy Namirembe[a], Shamira Nakalema[b], Stella Neema, Ph.D[d], Clare Tanton, Ph.D[c], Connie Alezuyo[e], Saidat Namuli Musoke[f], Belen Torondel, Ph.D[g], Suzanna C. Francis, Ph.D[c], David A Ross, Ph.D[h], Chris Bonell, Ph.D[c], Janet Seeley, Ph.D[a,c], *Helen A. Weiss, Ph.D[c,h].

[a] Medical Research Council / Uganda Virus Research Institute & London School of Hygiene & Tropical Medicine Uganda Research Unit, Plot 51-59 Nakiwogo Road Entebbe, Uganda, [b]WoMena Uganda, Plot 2150 Kaduyu Close, Ntinda-Kigowa Kampala, Uganda, [c] MRC Tropical Epidemiology Group, London School of Hygiene & Tropical Medicine, UK [d] Makerere University, College of Humanities and Social Science, Uganda, [e] Palm Tree Academy Uganda, Uganda, [f] Uganda Virus Research Institute Entebbe, Uganda [g] London School of Hygiene & Tropical Medicine, UK [h] Department of Maternal, Newborn, Child and Adolescent Health, World Health Organisation, Geneva, Switzerland.

*Corresponding author: Professor Helen A. Weiss.

Address: London School of Hygiene and Tropical Medicine, Keppel Street, London WC1E 7HT, United Kingdom

Email: helen.weiss@lshtm.ac.uk

Phone number: +44 207 927 2087

Word count: 3607 words

For peer review only - http://bmjopen.bmj.com/site/about/guidelines.xhtml

For peer review only

Abstract

Objectives: Menstrual health management (MHM) is a public health challenge and there is a lack of evidence to inform interventions. The objective of this study was to pilot-test an intervention to improve MHM and school attendance in Uganda, in preparation for a cluster-randomised trial.

Design: Longitudinal study with pre-post evaluation of a pilot intervention

Setting: Two secondary schools in Entebbe, Uganda

Participants: Of the 473 eligible students in Secondary 2 (S2) at baseline, 450 (95.1%; 232 girls and 218 boys) consented/assented. Overall, 369 students (188 girls; 81.0%; and 181 boys; 83.0%) participated in the endline survey.

Intervention: The intervention package comprised training teachers to improve delivery of government guidelines for puberty education, training in use of a menstrual kit (including re-usable pads) and pain management, a drama skit, provision of analgesics and improvements to school water and sanitation facilities.

Primary and secondary outcome measures: Feasibility and acceptability of delivering the intervention. Baseline and endline quantitative surveys were conducted, with qualitative interviews conducted at endline. School attendance was assessed using self-completed daily diaries among a nested closed cohort of 100 female students.

Results: The intervention was found to be acceptable and feasible to deliver from the qualitative and quantitative data. There were substantial reported improvements in menstrual-related stigma and anxiety, pain management, and some improvement in MHM knowledge of puberty and menstruation and Water, Sanitation and Hygiene (WASH) at endline compared with baseline, which were supported by qualitative findings. The diary data and qualitative data indicated a potential impact of the intervention on improving menstrual-related school absenteeism.

For peer review only - http://bmjopen.bmj.com/site/about/guidelines.xhtml

Conclusions: The pilot study showed that the multi-component MHM intervention was acceptable and feasible to deliver, and potentially effective on education and health outcomes. A cluster-randomised trial is needed to evaluate the intervention effects on MHM and school attendance.

Key words: Menstrual Health, Menstrual Hygiene, Adolescents, Education, School Attendance

For peer review only

Article Summary

Strengths and limitations of this study:

- The study population are students in peri-urban secondary schools, which is important given the recognised importance of girls' secondary education to future development, and evidence that school absenteeism due to menstruation is problematic in secondary school girls.
- The study evaluated a multi-component menstrual health intervention that addresses individual, behavioural and environmental barriers to good menstrual health and school attendance.
- This is the first menstrual health intervention to address menstrual pain – a major contributor to school absence in girls
- The intervention includes boys as well as girls, as sustainable changes in menstrual management depend on addressing stigma about menstruation.

For peer review only - http://bmjopen.bmj.com/site/about/guidelines.xhtml

For peer review only

For peer review only - http://bmjopen.bmj.com/site/about/guidelines.xhtml

INTRODUCTION

Poor menstrual health management (MHM) affects girls and women globally,[1-4] with many lacking knowledge, materials and facilities for safely managing menstruation without stigma.[2] Effective MHM interventions may lead to sustained benefits for health, productivity [3-5] and the environment [6] but there is little evidence to guide policies.[7-9] Systematic reviews identified nine MHM intervention trials with health, educational or psychosocial outcomes, with inconclusive results and high risk of bias.[5, 10]

Schools provide an important setting for addressing MHM challenges concerning stigma, lack of menstrual literacy and goods (menstrual products, improved water, sanitation and hygiene (WASH) facilities and pain management).[11] In 2014, the “MHM in Ten” initiative developed an agenda for addressing the barriers facing girls in schools in low-income contexts.[12] The first priority of the initiative is to generate rigorous evidence on whether improving MHM improves school attendance.[7, 8, 13]

The aim of this study was to pilot-test a multi-component school-based MHM intervention (“MENISCUS”) and to prepare for a future cluster-randomised trial (CRT) to evaluate the impact of the intervention on secondary school attendance, education, health and wellbeing outcomes in Wakiso District, Uganda.

METHODS

Study setting and participants

This pilot study was conducted in Entebbe Municipality in Wakiso District, Uganda. Entebbe has 13 registered secondary schools. Two day schools (one government and one private), both with students of low socio-economic status, were purposively selected. Eligible participants were all male and female students in the second school year (Secondary 2 (S2)).

For peer review only - http://bmjopen.bmj.com/site/about/guidelines.xhtml

Written informed consent was sought from students aged ≥18 years, and from the parents/caretakers of those aged <18 years, with student assent.

Intervention package

We had conducted a study in this setting to develop the intervention and found that effective MHM intervention needs to address stigma, education, attitudes and psychosocial well-being, and provide the goods defined above.[14] Following this, we finalised the pilot MENISCUS intervention, informed by social cognitive theory (SCT) which postulates that learning occurs in a social context and that personal factors, behavioural patterns and environmental aspects have bidirectional influences on one another.[15] Using the core constructs of SCT, we developed a theory of change (ToC) (Figure 1) with stakeholders to articulate the aims of the intervention: i) increase girls' self-efficacy to manage their menstruation (e.g. through provision of an MHM kit and pain-management options); ii) use observational learning to reinforce girls' learning and that of boys, teachers and parents to create a more supportive MHM environment (through drama); and iii) provide positive reinforcement for behaviour change (through improving WASH facilities and reduced leakage/pain as a result of better MHM).

The intervention was implemented in partnership with a local NGO, WoMena Uganda, and consisted of

i. Puberty education: WoMena Uganda worked with an educational consultant to train 30 teachers from the two schools to improve delivery of Ugandan Government guidelines for puberty education.

ii. Drama Skit: To address stigma, attitudes and psychosocial issues about menstruation, WoMena Uganda trained school teachers and students to develop and perform drama skits. Topics included managing pain, teasing, parental responsibilities for supporting girls with menstruation and tracking the menstrual cycle.

iii. Menstrual management kit: A kit, including a pack of 4 AFRipad reusable menstrual pads, a small towel, soap, water bottle, underwear and menstrual calendar, was distributed to

For peer review only - http://bmjopen.bmj.com/site/about/guidelines.xhtml

all girls in S2. WoMena Uganda taught girls about effective pain relief strategies such as stretching, using a hot-water bottle and trained teachers and prefects to deliver education sessions on menstruation to girls and boys, on MHM and use of the re-usable pads to girls only.

iv. Pain management: The research team supplied each girl with vouchers to exchange for up to six paracetamol tablets per month from the school nurse, senior teacher or a local pharmacy.

v. WASH facilities: The research team improved school WASH facilities: improving access to water close to the sanitation facilities e.g. provision of water drums and stands (one with clean water and one with soapy water), installing locks, repairing broken doors, providing bins and providing toilet-paper holders.

Study design and evaluation

The pilot study had a repeat cross-sectional design with a nested closed cohort. All participants were asked to self-complete a quantitative survey at baseline (October 2017) and endline (August 2018) on tablets using Open-Data-Kit software. Questions included knowledge of puberty and menstruation, perceptions of menstruation (using a 5-point Likert scale), menstrual management practices at last menstrual period (LMP), reported pain and pain management strategies, leakages during LMP, anxiety, behavioural and conduct problems, using the 25-item Strengths and Difficulties Questionnaire (SDQ).

The nested closed cohort comprised a random sub-sample of 50 girls per school assessing the feasibility of school attendance and menstruation patterns using self-completed daily diaries. Cohort participants were given a booklet each term for daily recording of school and class attendance, menstruation and pain.

Qualitative interviews were used to assess perceptions and acceptability of the intervention, and its perceived impact on school attendance at endline. In-depth interviews (IDIs) were

For peer review only - http://bmjopen.bmj.com/site/about/guidelines.xhtml

For peer review only

conducted with 20 female students sampled purposively (menstruating S2 girls with different levels of reported baseline school attendance), 10 teachers (6 females and 4 males) and 10 parents who had attended the drama performance. Four focus-group discussions (FGDs) were conducted (two with each sex). Semi-structured topic guides were used for both IDIs and FGDs. Interviewers were the same sex as respondents and interviews were digitally recorded with permission.

Research assistants conducted nine unannounced WASH assessment visits per school using a standardized checklist to assess availability, accessibility and functioning in terms of sanitary waste disposal, availability of water, soap, hand-washing facilities and toilet paper; cleanliness and privacy.

Data management and analysis

Electronically-captured data were exported to Stata version 15. The paper-based diary data and WASH checklist data were entered using Microsoft Access. For the survey data, the intervention effect on binary outcomes was estimated by comparing endline versus baseline measures, using adjusted odds ratios (AOR) and 95% confidence intervals (95%CI) estimated with random-effects logistic regression accounting for within-individual clustering. Effects on continuous outcomes were similarly assessed with random-effects linear regression to estimate the adjusted mean difference (AMD) and 95%CI. All analyses were adjusted for school, gender and age (<16, >16 years) as fixed effects. Moderation by school and age group were assessed using the likelihood ratio test. For the nested cohort, period-days were defined from the diary data as the days of menstruation (bleeding) plus the day prior to menstruation, with sensitivity analyses restricted to days of menstruation only.

Audio-recordings were transcribed verbatim and translated into English. Data were analysed using thematic content analysis, with two social scientists independently coding and checking

For peer review only - http://bmjopen.bmj.com/site/about/guidelines.xhtml

results. Key themes and subthemes related to the objectives of the qualitative research were organised in a matrix and discussed by the team for appropriate interpretation.

Patient and public involvement

Community groups were involved throughout the formative work, with stakeholders' workshops in August 2016 and October 2018, and a Theory of Change (ToC) workshop in April 2017. The ToC workshop included 35 invited participants (teachers, students and parents from the MENISCUS-1 and -2 schools, representatives from the Ministry of Education and Sports (MoES), Ministry of Health (MoH), the District Education Officer, Makerere University and NGOs working on menstrual health). In October 2018, we disseminated findings from MENISCUS-2 at a stakeholders' workshop, and elicited input into the future trial design. This meeting was attended by 60 participants, including representatives from MoES, MoH, Uganda National Council for Science and Technology (UNCST), Wakiso District Local Government Makerere University, AFRIpads, WoMena and Entebbe Municipal Council. MENISCUS-1 and 2 schools were represented by head teachers, students and parents.

RESULTS

Socio-demographic characteristics

Of the 473 eligible S2 students at baseline, 450 (95.1%; 232 girls and 218 boys) consented/assented. Overall, 369 students (188 girls; 81.0%; and 181 boys; 83.0%) participated in the endline survey. At baseline, the mean age was 15.4 years (SD 1.31; range 12-20) for girls and 16.2 (SD=1.5; range 13-21) for boys. Among the girls, 222 (95.7%) and 183 (97.3%) had started menstruating at baseline and endline respectively. The majority (n=361; 80.2%) were Christian and 198 (44.0%) were of Ganda ethnicity. About half (50.6%) of participants' mothers had post-primary education, as did 73.5% of participants' fathers (excluding 74 mothers and 88 fathers where this information was unknown). Relatively few participants (n=59; 13.1%) had running water at home, and 54 (12.0%) had a flush toilet in the home.

For peer review only - http://bmjopen.bmj.com/site/about/guidelines.xhtml

Feasibility and acceptability of implementing the intervention

There were high levels of uptake of the individual and behavioural intervention components (puberty education, drama skit, MHM kit, and pain management). Most students (81.3%) reported attending puberty-education sessions and 93.4% of girls reported receiving the MHM kit. Both schools performed the drama skit, and the qualitative interviews indicated that the skit increased MHM awareness and enabled some of the girls to talk about MHM with their parents, especially their fathers.

"When we all saw the skit, it gave us a starting point to initiate a discussion with our children. Even men got it" **(***Female Parent***,** *37 year old).*

The WASH component was challenging to implement. At baseline, there were no sanitary disposal bins, lockable toilet paper holders, or functional water drums at either school. Both schools received these items as part of the intervention. At endline, in the private school, all the girls' cubicles were functional, clean and with a lockable door versus only 33% in the Government school, where facilities were shared with the community. Despite this, most girls (81.4%) reported improvements in toilet facilities at endline, which improved their comfort managing menstruation.

"The school environment is now conducive … during our menstruation because the water is available, they give us water mixed with liquid soap; the toilet doors have locks, there is privacy, a person cannot interrupt you while changing the pad. Toilet paper is available whenever needed and the toilets are always clean." (Female student, 17 years old; Private school).

Knowledge, misconceptions and perceptions of puberty and menstruation

For peer review only - http://bmjopen.bmj.com/site/about/guidelines.xhtml

Knowledge of puberty and menstruation was poor at baseline and endline, especially among boys. The proportion answering all nine knowledge questions correctly increased from 11.6% to 23.9% (AOR=4.03, 95%CI 1.88-8.68) in girls, and from 4.1% to 12.7% (AOR=4.11, 95%CI 1.62-8.68-10.5) in boys (Table 1). There was also evidence of improvements in knowledge of the menstrual cycle, particularly among girls, although endline knowledge remained poor (29.3% of girls and 7.7% of boys answering all 3 questions correctly; Table 1).

The proportion of girls answering at least eight of nine questions on their perceptions about menstruation positively (i.e. questions about self-confidence, teasing and anxiety) increased from 12.2% at baseline to 27.9% at endline (AOR=3.62, 95%CI: 1.85-7.11; Table 2). The largest effects were reductions in the proportion of girls reporting teasing by boys about menstruation (14.4% to 8.7%, AOR=0.43, 95%CI 0.19-0.96), girls feeling anxious about the next period (58.6% to 34.4%, AOR=0.14, 95%CI 0.06-0.31) and girls avoiding physical activity during menstruation (47.8% to 25.7%; AOR=0.27, 95%CI 0.15-0.50). There was no evidence of moderation by age (Table 2) or school (results not shown).

In qualitative interviews, girls and teachers said that they thought that fear, myths and negative perceptions about menstruation were due to lack of knowledge. Participants suggested that the intervention improved knowledge about puberty, menstruation, and girls' accounts suggested a reduction of teasing by boys at school:

"Before MENISCUS, boys used to laugh at girls, for example when a girl stood up in class with her dress stained, boys would laugh at her but after MENISCUS training, they stopped laughing at girls and they now care about us" (Female student, 18 years old; Private school).

For peer review only - http://bmjopen.bmj.com/site/about/guidelines.xhtml

Table 1: Knowledge of puberty and menstruation, attitudes to menstruation and menstrual practices at baseline and endline, by gender

	Girls			Boys		
	Baseline (n=232)	Endline (n=188)	AOR (95% CI)	Baseline (n=218)	Endline (n=181)	AOR (95% CI)
All 9 knowledge questions correct[1]	27 (11.6%)	45 (23.9%)	4.03 (1.88-8.68)	9 (4.1%)	23 (12.7%)	4.11 (1.62-10.5)
Knowledge excluding fertility question	70 (30.2%)	120 (63.8%)	7.02 (3.67-13.45)	42 (19.3%)	73 (40.3%)	4.16 (2.15-8.04)
All 4 myth questions correct[2]	39 (16.8%)	77 (41.0%)	5.44 (2.82-10.50)	22 (10.1%)	44 (24.3%)	3.99 (2.00-8.04)
All 3 menstrual cycle questions[3]	12 (5.2%)	55 (29.3%)	19.45 (5.88-64.5)	4 (1.8%)	14 (7.7%)	3.95 (1.26-12.4)

[1] Knowledge questions were: i) Adolescence is the time between puberty and adulthood; ii) Changes in the body during puberty happen because of hormones; iii) The physical changes related to puberty usually start between 10 and 14 years of age in girls, and between 12 and 16 in boys; iv) Menstrual blood comes from the stomach where food is digested; v) Women stop menstruating after the age of about 45-50; vi) Menstruation in girls and women is normal; vii) Pregnant women menstruate; viii) When a girl gets her first period, her body is ready to have children; ix) During her period a girl can get pregnant
[2] Myth questions were: i) Painkillers cause problems having children; ii) When a girl has her period she is unclean; iii) Sanitary pads can cause sickness or infection; iv) It is healthy for a girl to run, dance or cycle during her period
[3] Menstrual cycle questions were: i) What is period blood; ii) How long does a period usually last; iii) How many days are there usually between periods?

For peer review only - http://bmjopen.bmj.com/site/about/guidelines.xhtml

Management of menstruation

The proportion of girls reporting using manufactured menstrual materials exclusively (i.e. reusable or disposable pads, tampons or menstrual cups) during their LMP increased from 73.0% at baseline to 89.1% at endline (AOR=4.14, 95%CI 2.04-8.40). At endline, most girls (n=155; 82.5%) reported using reusable pads during their LMP compared with 18.5% at baseline. There was some evidence of a decrease in reported leakage of blood through their clothing during LMP (47.4% at baseline vs 35.3% at endline; AOR=0.51, 95%CI 0.25-1.04), but no evidence of a difference in the proportion of girls reporting staining their underwear during their LMP (23.4% at baseline to 27.3% at endline (AOR=1.34, 95%CI 0.78-2.30). There was no evidence of effect-moderation by age or school (Table 2).

Knowledge and use of effective pain management methods

Most girls reported pain during menstruation at both baseline and endline (74.3% and 71.0%, respectively). Amongst these girls, there was evidence of an increase in the proportion who reported using painkillers during their LMP (46.7% at baseline to 60.8% at endline; AOR=1.87, 95%CI 1.05-3.34). Results were similar by school and age (Table 2). There was evidence of an increase in the proportion of girls who reported using ≥1 effective pain relief method during their LMP (49.7% at baseline to 77.8% at endline; AOR=4.24, 95%CI 2.16-8.33). Of the 232 girls who received a voucher for painkillers, 58 (25.0%) had redeemed 78 vouchers by endline: 77% used one voucher, 16% used two, and 7% used ≥2.

The qualitative findings confirmed that non-pharmacological methods of pain relief were popular and effective.

"They [MENISCUS team] taught us how to do exercises to relieve pain and it worked for me so, the last time, I didn't use painkillers; I managed my periods by doing exercises and using reusable pads MENISCUS had provided." (Female student, 14 years old; Government school).

For peer review only - http://bmjopen.bmj.com/site/about/guidelines.xhtml

Table 2: Reported perceptions of menstruation, menstrual management, pain and pain management among girls, by age at baseline

	**Age <16 years**			**Age > 16 years**		
	**Baseline (n=115)**	**Endline (n=51)**	**AOR (95% CI)**	**Baseline (n=107)**	**Endline (n=132)**	**AOR (95% CI)**
>8 of 9 positive perceptions of menstruation[1]	14 (12.2%)	16 (31.4%)	5.25 (1.10-25.19)	13 (12.2%)	35 (26.5%)	2.98 (1.29-6.86)
Used manufactured menstrual materials only at LMP	86 (74.8%)	46 (90.2%)	3.40 (1.06-10.9)	76 (71.0%)	117 (88.6%)	4.24 (1.68-10.78)
Leaked blood at LMP	37 (50.0%)	12 (42.9%)	0.60 (0.18-2.04)	37 (45.1%)	24 (32.4%)	0.58 (0.28-1.18)
Underwear stained at LMP	31 (27.0%)	16 (31.4%)	1.14 (0.45-2.87)	21 (19.6%)	34 (25.8%)	1.49 (0.73-3.06)
Knew >4 effective pain management methods	30 (24.2%)	37 (67.3%)	10.80 (3.48-33.56)	30 (27.8%)	97 (72.9%)	21.62 (5.44-84.93)
Reported pain at last period	79 (68.7%)	36 (70.6%)	1.00 (0.37-2.65)	86 (80.4%)	94 (71.2%)	0.54 (0.26-1.10)
Used >1 effective pain management method[2]	57 (72.2%)	34 (94.4%)	6.78 (1.49-30.8)	69 (80.2%)	85 (90.4%)	2.79 (0.95-8.21)
Used painkillers at LMP[3]	32 (40.5%)	21 (58.3%)	2.16 (0.91-5.11)	45 (52.3%)	58 (61.7%)	1.70 (0.74-3.92)
Used other effective methods[2]	39 (49.4%)	29 (80.6%)	4.22 (1.66-10.78)	43 (50.0%)	72 (76.6%)	6.83 (1.88-24.85)
Did nothing for pain at LMP[1]	20 (25.3%)	2 (5.6%)	0.17 (0.04-0.79)	17 (19.8%)	7 (7.5%)	0.18 (0.03-0.99)

For peer review only

[1] Perception questions were i) I prefer staying at home during my period rather than going to school; ii) I worry about being teased during my period; iii) During my period I feel less self-confident than during other days; iv) During my period I avoid physical activity (e.g. walking, running); v) I feel anxious about having my next period; vi) Boys tease me about my period vii) Girls tease me about my period; viii) I feel comfortable to talk to other girls at school about my period; ix) If I had a problem with managing my period, I would talk to another girl about it

[2] Effective methods (painkiller, drinking water, using hot water bottle, exercise, relaxing, foods with lots of water).

[3] Among those with pain at LMP

Psychosocial well-being

The mean SDQ-25 score in girls decreased from 10.3 to 9.2 (p=0.006, adjusted for age and school), indicating improved behaviour and conduct. No decrease was seen among boys, in line with the ToC (9.88 at baseline vs 9.91 at endline; p=0.98).

Menstruation and school absence

In the cross-sectional surveys, a similar proportion of girls reported missing at least one school day due to menstruation in Term 2, 2017 (pre-baseline) and Term 2, 2018 (endline) (32.0% vs 32.8%; OR=0.97, 95%CI 0.57-1.66). Among the 100 cohort participants, data were collected from 81 students at endline. The AOR associated with missing school on period-days compared to non-period days decreased from OR=2.17 (95%CI 1.61-2.92) at baseline to OR=1.29 (95%CI 1.02-1.63) at endline (p-value for moderation =0.01; Table 3). Results were similar for class attendance (Table 3). The direct observation checks found high correlation with the self-completed diaries. Girls were seen on 328/330 (99.4%) of days when their diary stated they were present and were not seen on all 37 days when the diary stated that they were absent. School registers were incomplete and rarely completed by teachers.

Table 3: School and class attendance at baseline and endline among nested cohort participants, by period-day status

	**Term 3 2017 (baseline) 99 girls**		**Term 2 2018 (end-line) 81 girls**	
	**Non period-day**	**Period-day[1]**	**Non period-day**	**Period-day**
Number of days	2625	554	3829	787
N (%) not attending full day of school	8.5%	14.6%	11.6%	14.2%
**Odds ratio (95% CI)[2]**	OR=2.17 (1.61-2.92)		OR=1.29 (1.02-1.63)	
N (%) not attending all classes	11.4%	19.9%	17.3%	20.5%
Odds ratio (95% CI)	OR=2.12 (1.64-2.74)		OR=1.26 (1.04-1.54)	

[1] Includes days of menses plus day prior to menses (results similar when restricted to days of menses) obtained from daily diaries for 9 months
[2] Obtained from random effects logistic regression, allowing for within-girl clustering

For peer review only - http://bmjopen.bmj.com/site/about/guidelines.xhtml

The qualitative interviews suggested that girls were more likely to attend school during menstruation at endline than at baseline, with reasons including the training on pain management, tracking their menstrual cycle, and having re-usable pads.

"Some girls totally don't come to school every menstruation period. However, in our class, missing school is now rare because we have learnt how to manage the periods, pain and we have what to use. We now also know our cycle" (Girls FGD; Government school).

"Before MENISCUS study gave us diaries, I didn't know how to track my days. I used to come to school and my uniform would get soiled. Sometimes, I could ask for a pass out to go back home during my periods. […] But when MENISCUS came, they gave us diaries, I started filling them in and now I know my days; I come to school prepared." (Female, 17 year old; Private school).

DISCUSSION

To our knowledge, this is the first MHM intervention to be pilot-tested that focused equally on health promotion (psychosocial issues, stigma and knowledge) and goods (provision of pads and analgesics, and improved WASH facilities).[16]

Feasibility and accessibility of implementing the intervention

The qualitative and quantitative data showed that the intervention was feasible and acceptable to schools and stakeholders. A detailed process evaluation will be published separately. The main challenge with implementation was maintenance of the WASH component. To improve this component in a future trial, we will ask schools to establish an MHM Leadership Group (to include teachers, parents and students) responsible for ensuring each intervention element is delivered, maintained and sustained by the schools. Another challenge was that we did not

For peer review only - http://bmjopen.bmj.com/site/about/guidelines.xhtml

obtain ethical approval to offer girls a menstrual cup, due to concerns about whether cup insertion could lead to damage of the hymen and affect a girl's virginity. Further activities to inform parents about menstrual cups and discuss concerns are underway.

Potential intervention effect on knowledge, menstrual practices and school attendance

The study was designed to assess feasibility and accessibility of implementing the intervention, rather than assess the effectiveness. Although the findings suggest a potential intervention effect on education and health outcomes, these results need to be interpreted cautiously as the improvements reported may be partly due to i) older age at endline – there may have been improvements in knowledge and self-confidence in managing menstruation due to a cohort effect; ii) reporting biases - the outcome assessment team were involved with implementation and this may have caused social desirability bias affecting responses on attitudes or behaviours following the intervention; iii) biases due to the lack of a comparison group, and the endline survey being conducted in a different term to baseline. For example, girls reported higher levels of school attendance overall at baseline than at endline, and this may be due to the baseline being conducted during an examination term.

The study highlighted poor knowledge of menstruation, possibly due to cultural taboos and norms,[14, 17] and lack of knowledge among students [18] parents and teachers hindering discussion of puberty and menstruation.[14, 19-21] Three intervention components addressed knowledge (puberty education, menstrual-management kit training, and drama skit). To address stigma and effect a school-wide change in attitudes, the education and drama sessions included boys. This pilot study showed that girls were less anxious about menstruation, and reported less teasing by boys after the intervention, supporting the theory of change, and previous qualitative work from Ghana that indicated reductions in shame and improvements in self-confidence following provision of pads and puberty education.[22]

For peer review only - http://bmjopen.bmj.com/site/about/guidelines.xhtml

At endline, compared with baseline, more girls reported use of only manufactured materials during their LMP, and using these correctly. The high uptake of reusable pads was expected, as these were provided free to all girls, but the continued use over follow-up, and favourable qualitative reports, shows that pads were acceptable. Similar findings on correct use have been seen in another study in Uganda where 98% of girls reported washing and using soap for their AFRIpads [16] and in India,[23] where a school-based health-education intervention led to improvements in washing clothes with soap, drying them in the sun and safe disposal.

Previous studies show inconsistent evidence for a relationship between menstruation and school absenteeism.[10, 14, 24-31] Documenting school attendance is often challenging in LMICs due to incomplete or inaccurate school registers.[5, 14] Our diaries were popular, and correlated well with direct observation. The improved school attendance during menstruation in this study was attributed to improved pain management, knowledge to track menstrual cycles, and the provision of reusable pads in qualitative interviews.

Innovative aspects of the MENISCUS intervention

1) Multi-component focus. Past and ongoing studies tend to focus on single-component interventions.[5, 27] This study indicates synergies between the intervention elements e.g. the drama skit reinforces puberty knowledge, reduces stigma and engages parents and boys. An emerging view, aligned with our findings, is that poor MHM is a social problem and to be effective, MHM interventions need to address the broader issues of menstrual stigma and literacy as well as the provision of menstrual products or improving WASH facilities.[7] The multi-component approach addresses both individual, behavioural and environmental barriers to good MHM and school attendance.

2) Pain management. Menstrual pain is a major contributor to school absence in girls in many settings.[14, 17, 32, 33] This is the first MHM intervention to address this issue and we found substantial improvements in pain management.[14] Further research is needed on this topic

For peer review only - http://bmjopen.bmj.com/site/about/guidelines.xhtml

as misconceptions about use of painkillers are common in Uganda [14] and elsewhere.[34, 35]

3) Inclusion of boys. Most MHM interventions focus only on girls, yet sustainable changes in MHM depend on addressing stigma about menstruation. This study highlighted the importance of including boys which is aligned with SCT (i.e. the intervention provides positive reinforcement for behavioural change by improving the school environment). The focus on boys as well as girls is supported by WHO framework for Health Promoting Schools [36] and is aligned with a report commissioned by the Gates Foundation on Menstrual Health and Gender Equity.[37]

4) Urban secondary school setting. Most intervention studies to date have been in primary schools, and in rural areas.[16, 32] MHM and school attendance is an issue in the peri-urban area in Entebbe where most girls can afford to buy disposable pads for some of the time.[14] A focus on MHM in secondary schools is important given the recognised importance of girls' secondary education to future development,[38] and evidence that school absenteeism due to menstruation is problematic in secondary school girls.[14, 39]

CONCLUSION

This pilot study showed that the MHM intervention was acceptable and feasible to implement. There were substantial reported improvements in stigma and anxiety, pain management, and some improvement in MHM knowledge of puberty and menstruation and WASH following a multi-component intervention. Based on SCT, we anticipate the results to be generalizable to other similar contexts with relatively low levels of MHM knowledge, stigma about discussing menstruation, and poor school WASH facilities. A phase-III trial is warranted to evaluate the impact of the intervention on school attendance, and on health, well-being and educational outcomes for definitive results to drive forward policy changes.

For peer review only - http://bmjopen.bmj.com/site/about/guidelines.xhtml

Statements:

Author contributions: HAW, LH, BT, DAR, JS, CB contributed to the overall conception and design of the study. CK and HAW wrote the first draft of the manuscript. All authors contributed to the interpretation of results and drafting of the manuscript. HAW and CT did the statistical analyses. All authors take responsibility for the integrity of the data and the accuracy of the data analysis. All authors read and approved the final manuscript. HAW is the guarantor.

Funding statement: This work was supported by a United Kingdom (UK) Department for International Development (DFID), the National Institute for Health Research (NIHR), UK Medical Research Council (MRC), and Wellcome Trust Joint Global Health Trials Development Grant (number MR/P020283/1).

Acknowledgements: The authors would like to thank the study participants, contact teachers in the two schools, and the Ministry of Education and Sports and Ministry of Health Uganda for their support.

Competing Interests: None declared

Disclaimer: The content is solely the responsibility of the authors and does not necessarily represent the official views of the funders.

Ethical approval: Ethics approval was received from the UVRI Research Ethics Committee, the Uganda National Council for Science and Technology (UNCST) and the London School of Hygiene and Tropical Medicine.

Data sharing statement: Data are available from LSHTM Data Compass on request.

Patient request for publication: Not required

For peer review only

REFERENCES

[1] Das P, Baker KK, Dutta A et al. Menstrual Hygiene Practices, WASH Access and the Risk of Urogenital Infection in Women from Odisha, India. PloS One 2015;10:e0130777.
[2] Sommer M, Caruso BA, Sahin M et al. A Time for Global Action: Addressing Girls' Menstrual Hygiene Management Needs in Schools. PLoS medicine 2016;13:e1001962.
[3] Sommer M, Sahin M. Overcoming the taboo: advancing the global agenda for menstrual hygiene management for schoolgirls. American journal of public health 2013;103:1556-9.
[4] Sommer M, Hirsch JS, Nathanson C, Parker RG. Comfortably, Safely, and Without Shame: Defining Menstrual Hygiene Management as a Public Health Issue. American journal of public health 2015;105:1302-11.
[5] Sumpter C, Torondel B. A systematic review of the health and social effects of menstrual hygiene management. PloS one 2013;8:e62004.
[6] Elledge MF, Muralidharan A, Parker A et al. Menstrual Hygiene Management and Waste Disposal in Low and Middle Income Countries-A Review of the Literature. 2018;15.
[7] Bobel C. The Managed Body: Developing Girls and Menstrual Health in the Global South: Palgrave Macmillan; 2018.
[8] Hennegan J. Menstrual Hygiene Management and Human Rights: The Case for an Evidence-Based Approach. Women's Reproductive Health 2017;4:212-31.
[9] Phillips-Howard PA, Caruso B, Torondel B et al. Menstrual hygiene management among adolescent schoolgirls in low- and middle-income countries: research priorities. Global health action 2016;9:33032.
[10] Hennegan J, Montgomery P. Do Menstrual Hygiene Management Interventions Improve Education and Psychosocial Outcomes for Women and Girls in Low and Middle Income Countries? A Systematic Review. PloS one 2016;11:e0146985.
[11] United Nations International Children's Emergency Fund, World Health Organisation. Drinking Water, Sanitation and Hygiene in Schools: Global baseline report 2018. New York: . 2018.
[12] United Nations International Children's Emergency Fund. MHM in Ten; Advancing the MHM agenda in schools. Third annual meeting co-hosted by Columbia University and UNICEF New York. http://www.ungei.org/2016_MHM_in_Ten_Meeting_Report.pdf. 2016.
[13] UNICEF. MHM in Ten: Advancing the MHM agenda in WASH in Schools. New York 2014.
[14] Miiro G, Rutakumwa R, Nakiyingi-Miiro J et al. Menstrual health and school absenteeism among adolescent girls in Uganda (MENISCUS): a feasibility study. BMC women's health 2018;18:4.
[15] Bandura A. Health promotion by social cognitive means. Health Educ Behav 2004;31:143-64.
[16] Hennegan J, Dolan C, Wu M, et al. Schoolgirls' experience and appraisal of menstrual absorbents in rural Uganda: a cross-sectional evaluation of reusable sanitary pads. Reproductive health 2016;13:143.
[17] UNICEF Western and Central A firica Regional Office. Menstrual Hygiene in Schools in 2 countries of Francophone West Africa; Burkina Faso and Niger Case Studies. 2013.
[18] Shanbhag D, Shilpa R, D'Souza N et al. Perceptions regarding menstruation and Practices during menstrual cycles among high school going adolescent girls in resource limited settings around Bangalore City, Karnataka, India. . Int J Collab Res Inter Med Public Health 2012;4:1353–62.
[19] Chandra-Mouli V, Patel SV. Mapping the knowledge and understanding of menarche, menstrual hygiene and menstrual health among adolescent girls in low- and middle-income countries. Reproductive health 2017;14:30.
[20] McMahon SA, Winch PJ, Caruso BA et al. 'The girl with her period is the one to hang her head' Reflections on menstrual management among schoolgirls in rural Kenya. BMC international health and human rights 2011;11:7.

For peer review only - http://bmjopen.bmj.com/site/about/guidelines.xhtml

[21] Bosch AM, Hutter I, van Ginneken JK. Perceptions of adolescents and their mothers on reproductive and sexual development in Matlab, Bangladesh. International journal of adolescent medicine and health 2008;20:329-42.
[22] Dolan CS, Ryus CR, Dopson S, et al. A blind spot in girls' education: menarche and its webs of exclusion in Ghana. Journal of International Development 2013;26:643±57.
[23] Nemade D, Anjenaya S, Gujar R. Impact of health education on knowledge and practices about menstruation among adolescent school girls of Kalamboli, Navi-Mumbai. . Health Popul Perspect Issues 2009;32:167–75.
[24] Grant MJ, Lloyd CB, Mensch BS. Menstruation and School Absenteeism: Evidence from Rural Malawi. Comparative education review 2013;57:260-84.
[25] van Eijk AM, Sivakami M, Thakkar MB et al. Menstrual hygiene management among adolescent girls in India: a systematic review and meta-analysis. BMJ open 2016;6:e010290.
[26] Oster E, Thornton R. Menstruation, Sanitary Products, and School Attendance: Evidence from a Randomized Evaluation. American Economic Journal: Applied Economics 2011;3:91-100.
[27] Phillips-Howard PA, Nyothach E, Ter Kuile FO et al. Menstrual cups and sanitary pads to reduce school attrition, and sexually transmitted and reproductive tract infections: a cluster randomised controlled feasibility study in rural Western Kenya. BMJ open 2016;6:e013229.
[28] Tegegne TK, Sisay MM. Menstrual hygiene management and school absenteeism among female adolescent students in Northeast Ethiopia. BMC public health 2014;14:1118.
[29] Montgomery P, Ryus CR, Dolan CS et al. Sanitary pad interventions for girls' education in Ghana: a pilot study. PloS one 2012;7:e48274.
[30] Wilson E, Reeve J, Pitt A. Education. Period. Developing an acceptable and replicable menstrual hygiene intervention. Development in Practice 2014;24:63±80.
[31] Scott L, Dopson S, Montgomery P et al. Impact of Providing Sanitary Pads To Poor Girls in Africa. University of Oxford. 2009.
[32] Boosey R, Prestwich G, Deave T. Menstrual hygiene management amongst schoolgirls in the Rukungiri district of Uganda and the impact on their education: a cross-sectional study. The Pan African medical journal 2014;19:253.
[33] Crofts T, J. F. Menstrual hygiene in Ugandan schools: an investigation of low-cost sanitary pads. J Water Sanitation Hyg Dev 2012;2:50–8.
[34] Kontogiorgis C, Nena E, Berberoglou E et al. Estimating Consumers' Knowledge and Attitudes Towards Over-The-Counter Analgesic Medication in Greece in the Years of Financial Crisis: The Case of Paracetamol. Pain and therapy 2016;5:19-28.
[35] Palos GR, Mendoza TR, Cantor SB et al. Perceptions of analgesic use and side effects: what the public values in pain management. Journal of pain and symptom management 2004;28:460-73.
[36] Langford R, Bonell C, Jones H et al. The World Health Organization's Health Promoting Schools framework: a Cochrane systematic review and meta-analysis. BMC Public Health 2015;15:130.
[37] Geertz A, Iyer L, Kasen P et al. An opportunity to address menstrual health and gender equity. In: FSG, editor.: Bill & Melinda Gates Foundation; May 2016.
[38] Wodon Quentin T, Montenegro Claudio E, Nguyen Hoa, et al. Missed Opportunities : The High Cost of Not Educating Girls (English). The Cost of Not Educating Girls Series. Washington, D.C. : World Bank Group. http://documents.worldbank.org/curated/en/775261531234655903/Missed-Opportunities-The-High-Cost-of-Not-Educating-Girls. 2018.
[39] Vashisht A, Pathak R, Agarwalla R et al. School absenteeism during menstruation amongst adolescent girls in Delhi, India. Journal of family & community medicine 2018;25:163-8.

For peer review only - http://bmjopen.bmj.com/site/about/guidelines.xhtml

Statements:

For peer review only

For peer review only - http://bmjopen.bmj.com/site/about/guidelines.xhtml

Figure 1

Figure 1: Theory of change for the MENISCUS intervention

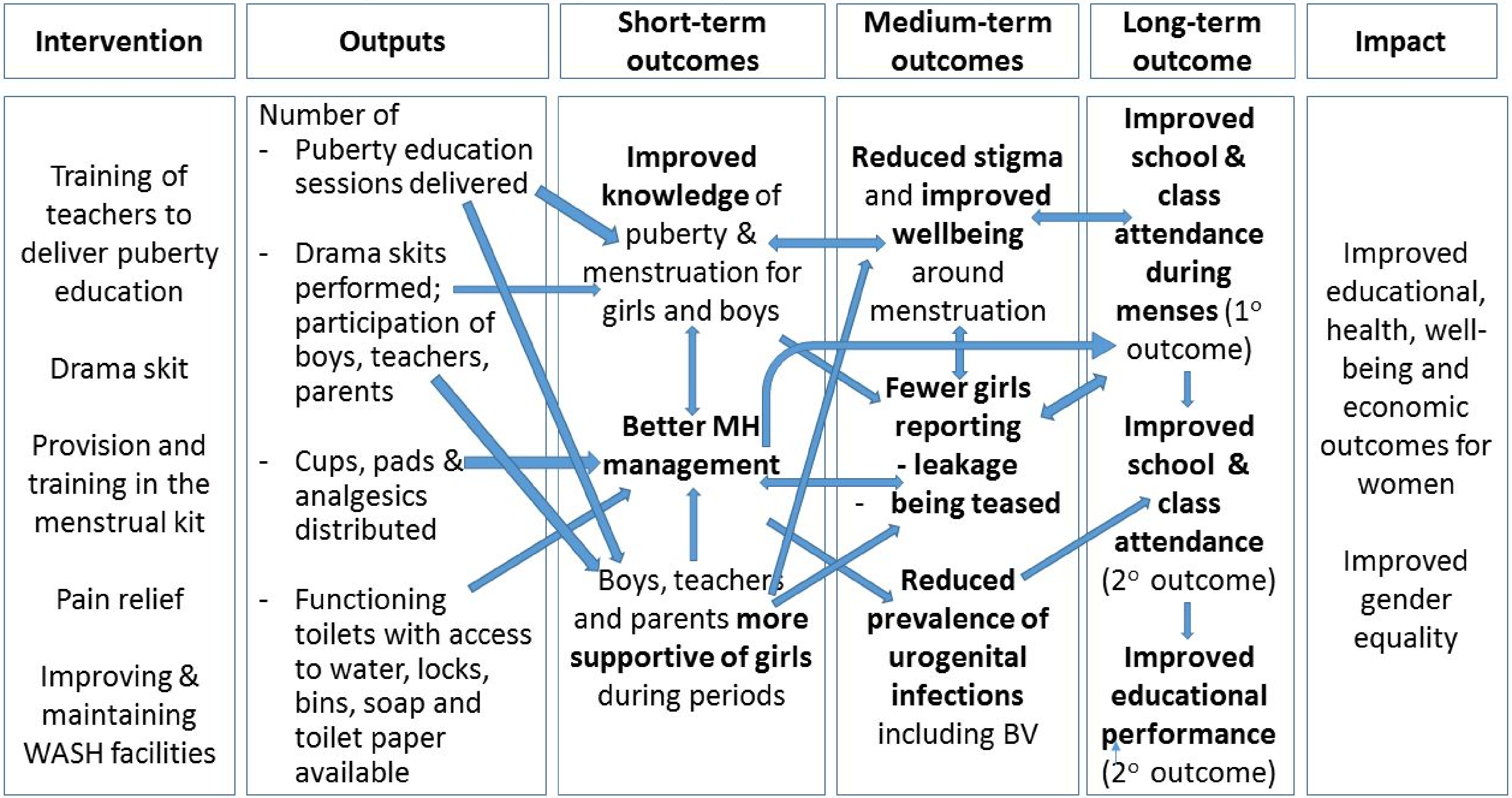

BMJ Open

Menstrual health intervention and school attendance in Uganda (MENISCUS-2): A pilot intervention study

Journal:	*BMJ Open*
Manuscript ID	bmjopen-2019-031182.R1
Article Type:	Original research
Date Submitted by the Author:	20-Aug-2019
Complete List of Authors:	Kansiime, Catherine; Medical Research Council / Uganda Virus Research Institute & London School of Hygiene & Tropical Medicine Uganda Research Unit<br>Hytti, Laura; WoMena Uganda<br>Nalugya, Ruth; Medical Research Council / Uganda Virus Research Institute & London School of Hygiene & Tropical Medicine Uganda Research Unit<br>Nakuya, Kevin; Medical Research Council / Uganda Virus Research Institute & London School of Hygiene & Tropical Medicine Uganda Research Unit<br>Namirembe, Prossy; Medical Research Council / Uganda Virus Research Institute & London School of Hygiene & Tropical Medicine Uganda Research Unit<br>Nakalema, Shamirah; WoMena Uganda<br>Neema, Stella; Makerere University, College of Humanities and Social Science<br>Tanton, Clare; London School of Hygiene and Tropical Medicine, Epidemiology and Population Health<br>Alezuyo, Connie; Palm Tree Academy Uganda<br>Namuli Musoke, Saidat; Uganda Virus Research Institute<br>Torondel, Belen; London School of Hygiene and Tropical Medicine, Faculty of Infectious and Tropical Diseases<br>Francis, Suzanna ; London School of Hygiene and Tropical Medicine, MRC Tropical Epidemiology Group, Faculty of Epidemiology and Population Health<br>Ross, David; World Health Organization, Department of Maternal, Newborn, Child and Adolescent Health<br>Bonell, Christopher; London School of Hygiene and Tropical Medicine, Public Health and Policy<br>Seeley, Janet; London School of Hygiene and Tropical Medicine Faculty of Public Health and Policy; Medical Research Council / Uganda Virus Research Institute & London School of Hygiene & Tropical Medicine Uganda Research Unit<br>Weiss, Helen; London School of Hygiene and Tropical Medicine, MRC Tropical Epidemiology Group, Faculty of Epidemiology and Population Health
**Primary Subject Heading**:	Global health
Secondary Subject Heading:	Epidemiology

Keywords:	Menstrual Health, School attendance, Menstrual Hygiene, Adolescent Health, Education

SCHOLARONE™
Manuscripts

I, the Submitting Author has the right to grant and does grant on behalf of all authors of the Work (as defined in the below author licence), an exclusive licence and/or a non-exclusive licence for contributions from authors who are: i) UK Crown employees; ii) where BMJ has agreed a CC-BY licence shall apply, and/or iii) in accordance with the terms applicable for US Federal Government officers or employees acting as part of their official duties; on a worldwide, perpetual, irrevocable, royalty-free basis to BMJ Publishing Group Ltd ("BMJ") its licensees and where the relevant Journal is co-owned by BMJ to the co-owners of the Journal, to publish the Work in this journal and any other BMJ products and to exploit all rights, as set out in our licence.

The Submitting Author accepts and understands that any supply made under these terms is made by BMJ to the Submitting Author unless you are acting as an employee on behalf of your employer or a postgraduate student of an affiliated institution which is paying any applicable article publishing charge ("APC") for Open Access articles. Where the Submitting Author wishes to make the Work available on an Open Access basis (and intends to pay the relevant APC), the terms of reuse of such Open Access shall be governed by a Creative Commons licence – details of these licences and which Creative Commons licence will apply to this Work are set out in our licence referred to above.

Other than as permitted in any relevant BMJ Author's Self Archiving Policies, I confirm this Work has not been accepted for publication elsewhere, is not being considered for publication elsewhere and does not duplicate material already published. I confirm all authors consent to publication of this Work and authorise the granting of this licence.

For peer review only

Menstrual health intervention and school attendance in Uganda (MENISCUS-2): A pilot intervention study

Catherine Kansiime, Ph.D[a], Laura Hytti, MMSc[b], Ruth Nalugya[a], Kevin Nakuya[a], Prossy Namirembe[a], Shamirah Nakalema[b], Stella Neema, Ph.D[d], Clare Tanton, Ph.D[c], Connie Alezuyo[e], Saidat Namuli Musoke[f], Belen Torondel, Ph.D[g], Suzanna C. Francis, Ph.D[c], David A Ross, Ph.D[h], Christopher Bonell, Ph.D[c], Janet Seeley, Ph.D[a,c], *Helen A. Weiss, Ph.D[c,h].

[a] Medical Research Council / Uganda Virus Research Institute & London School of Hygiene & Tropical Medicine Uganda Research Unit, Plot 51-59 Nakiwogo Road Entebbe, Uganda, [b]WoMena Uganda, Plot 2150 Kaduyu Close, Ntinda-Kigowa Kampala, Uganda, [c] MRC Tropical Epidemiology Group, London School of Hygiene & Tropical Medicine, UK [d] Makerere University, College of Humanities and Social Science, Uganda, [e] Palm Tree Academy Uganda, Uganda, [f] Uganda Virus Research Institute Entebbe, Uganda [g] London School of Hygiene & Tropical Medicine, UK [h] Department of Maternal, Newborn, Child and Adolescent Health, World Health Organisation, Geneva, Switzerland.

*Corresponding author: Helen A. Weiss. Present address: London School of Hygiene and Tropical Medicine, Keppel Street, London WC1E 7HT, United Kingdom *Email:* *helen.weiss@lshtm.ac.uk,* Phone number: +44 207 927 2087

For peer review only

Abstract

Objectives: Achieving good menstrual health and hygiene (MHH) is a public health challenge and there is a lack of evidence to inform interventions. The aim of this study was to pilot-test an intervention to improve MHH and school attendance in Uganda, in preparation for a future cluster-randomised trial.

Design: Longitudinal study with pre-post evaluation of a pilot intervention

Setting: Two secondary schools in Entebbe, Uganda

Participants: Of the 473 eligible students in Secondary 2 (S2) at baseline, 450 (95.1%; 232 girls and 218 boys) consented/assented. 369 students (188 girls; 81.0%; and 181 boys; 83.0%) participated in the endline survey.

Intervention: The intervention comprised training teachers to improve delivery of government guidelines for puberty education, training in use of a menstrual kit and pain management, a drama skit, provision of analgesics and improvements to school Water and Sanitation Hygiene (WASH) facilities.

Primary and secondary outcome measures: Feasibility and acceptability of delivering the intervention. Baseline and endline quantitative surveys were conducted, with qualitative interviews conducted at endline. School attendance was assessed using self-completed daily diaries among a nested closed cohort of 100 female students.

Results: The intervention was acceptable and feasible to deliver, and there were substantial reported improvements in MHH. The proportion of girls reporting anxiety about next period decreased from 58.6% to 34.4%, and reported use of effective pain management increased from 76.4% to 91.4%. Most girls (81.4%) reported improved school toilet facilities, which improved their comfort managing menstruation. The diary data and qualitative data indicated a potential impact of the intervention on improving menstrual-related school absenteeism.

For peer review only

Conclusions: The pilot study showed that the multi-component MHM intervention was acceptable and feasible to deliver, and potentially effective in improving menstruation knowledge and management. A cluster-randomised trial is needed to evaluate rigorously the intervention effects on MHM and school attendance.

Strengths and limitations of the study:

- The study evaluated a multi-component menstrual health intervention that addresses individual, behavioural and environmental barriers to good menstrual health and school attendance.
- The study population are students in peri-urban secondary schools, which is important given the recognised importance of girls' secondary education to future development, and evidence that school absenteeism due to menstruation is problematic for secondary school girls.
- This is the first menstrual health intervention to address menstrual pain – a major contributor to school absence in girls
- The intervention includes boys as well as girls, as sustainable changes in menstrual management depend on addressing stigma about menstruation.
- The conclusions are limited due to the lack of a control group, which means that the improvements seen in knowledge, school attendance and wellbeing may reflect the girls being older at endline or differences in attendance in different terms.

For peer review only

INTRODUCTION

Many girls and women globally lack the knowledge, materials and facilities for safely managing menstruation without stigma.[1-4] Adequate menstrual hygiene management (MHM) is defined as i) using clean menstrual management material to absorb or collect menstrual blood, ii) that can be changed in privacy as often as necessary for the duration of a menstrual period, iii) using soap and water for washing the body as required, iv) having access to safe and convenient facilities to dispose of used menstrual management materials, v) understanding the basic facts linked to the menstrual cycle and vi) how to manage it with dignity and without discomfort or fear.[5] A recent systematic review of menstrual experiences among women and adolescent girls globally highlighted that interventions must address a broad range of issues including addressing stigma, knowledge, social support, restrictive behavioural expectations, and the physical and economic environment.[6] Aligned with this, the term menstrual health and hygiene (MHH) encompasses both MHM and the broader systemic factors that link menstruation with health, well-being, gender equality, education, equity, empowerment, and rights.[5]

Effective MHH interventions may lead to sustained benefits for health, productivity [3, 4, 7] and the environment [8] but there has been little rigorous evaluation of interventions to guide policies.[9-11] Schools provide an important setting for addressing MHH challenges concerning stigma, lack of menstrual literacy and goods (menstrual products, improved water, sanitation and hygiene (WASH) facilities and pain management).[12] In 2014, the “MHM in Ten” initiative developed an agenda for addressing the barriers facing girls in schools in low-income contexts.[13] The first priority of the initiative is to generate rigorous evidence on whether improving MHM improves school attendance.[9, 10, 14] A systematic review published in 2016 [15] concluded there was promising evidence of the effectiveness of MHH interventions on educational outcomes, but insufficient evidence of effect due to a small number of trials (N=8), a high risk of bias, and substantial heterogeneity. An earlier systematic review [7] of intervention and observational studies found that of the 11 studies (only one RCT)

For peer review only - http://bmjopen.bmj.com/site/about/guidelines.xhtml

For peer review only

investigating the association between MHM and urogenital infections, seven found an increased risk associated with "worse" MHM (defined differently for each study but generally meaning not using disposable sanitary pads), one found the reverse to be true (an increased risk from using disposable sanitary pads), and three found no association.

The aim of this study was to pilot-test a multi-component school-based MHH intervention ("MENISCUS") and to prepare for a future cluster-randomised trial (CRT) which will evaluate the impact of the intervention on secondary school attendance, performance, menstruation knowledge, health and wellbeing outcomes in Uganda. Formative work in Wakiso District, Uganda, showed that secondary school girls reported substantial embarrassment and fear of teasing related to menstruation, reporting that this, together with menstrual pain and lack of effective materials for menstrual hygiene management, led to school absenteeism, especially in schools with low socio-economic catchment populations.[16] The issue is recognised by the Ugandan Government, which has the political will to improve MHM.[17] However the Government MHM guidelines for schools are not consistently implemented [18] for reasons including lack of funding, unclear roles and responsibilities for MHM, with issues largely left to the senior teachers.

The objectives of the pilot study are to: i) Pilot a combined package of MHH intervention elements developed in the formative work, delivered to a whole secondary school year for nine months; ii) Measure the uptake and assess perceptions of each element of the intervention package, and the package as a whole; iii) Pilot the use of daily diaries to estimate school attendance, and compare attendance with estimates using registers, observation visits and retrospective self-report; iv) Estimate school retention over a 9 month period; and v) Estimate the sample size for a future trial.

For peer review only - http://bmjopen.bmj.com/site/about/guidelines.xhtml

METHODS

Study setting and participants

This pilot study was conducted in Entebbe Municipality in Wakiso District, Uganda. Entebbe has 13 registered secondary schools. Two day schools (one government and one private), both with students of low socio-economic status (SES), were purposively selected. In this context, low SES schools are characterised by parents with low income, lower education achievement and under resourced in terms of low grade of teachers and facilities such as infrastructure. Eligible participants were all male and female students in the second school year (Secondary 2 (S2)). Written informed consent was sought from students aged $\geq$18 years, and from the parents/caretakers of those aged <18 years, with student assent. We also received written informed consent from teachers and parents/caretakers participating in interviews. Participation was voluntary, and confidentiality was ensured by conducting interviews in a private setting and keeping the data collected on secure servers without identifying information.

Intervention package

We previously conducted a study in this setting to develop the intervention and found that an effective MHH intervention needs to address stigma, education, attitudes and psychosocial well-being, and provide the goods defined above.[16] Following this, we finalised the pilot MENISCUS intervention, informed by social cognitive theory (SCT) which postulates that learning occurs in a social context and that personal factors, behavioural patterns and environmental aspects have bidirectional influences on one another.[19] Using the core constructs of SCT, we developed a Theory of Change (ToC) (Figure 1) with stakeholders to articulate the aims of the intervention: i) increase girls' self-efficacy to manage their menstruation (e.g. through provision of an MHM kit and pain-management options); ii) use observational learning to reinforce girls' learning and that of boys, teachers and parents to create a more supportive MHM environment (through drama); and iii) provide positive

For peer review only - http://bmjopen.bmj.com/site/about/guidelines.xhtml

reinforcement for behaviour change (through improving WASH facilities and reduced leakage/pain as a result of better MHM).

The intervention was implemented in partnership with a local NGO, WoMena Uganda, and consisted of:

i. Puberty education: We used a Training of Trainers (ToT) model in which WoMena Uganda worked with an educational consultant and her assistant to train 30 teachers from the two schools to improve delivery of Ugandan Government guidelines for puberty education to their students. The trained teachers were expected to develop a plan for delivering puberty training in their school and deliver the specified number of sessions to both male and female students.
ii. Drama Skit: To address stigma, attitudes and psychosocial issues about menstruation, WoMena Uganda facilitated students, with support from the school drama teacher, to develop and perform drama skits. Topics included managing pain, teasing, parental responsibilities for supporting girls with menstruation and tracking the menstrual cycle.
iii. Menstrual management kit: A kit, including a pack of 4 AFRipads reusable menstrual pads, a small towel, soap, water bottle, underwear, a mirror and menstrual calendar, was distributed to all girls in S2. WoMena Uganda trained teachers and prefects to deliver education sessions on menstruation to girls and boys on MHH, pain relief strategies such as stretching, using a hot-water bottle and use of the re-usable pads to girls only.
iv. Pain management: The research team supplied each girl with vouchers to exchange for up to six paracetamol tablets per month from the school nurse, senior teacher or a local pharmacy.
v. WASH facilities: The research team improved school WASH facilities by improving access to water close to the sanitation facilities e.g. provision of water drums and stands (one with clean water and one with soapy water), installing locks, repairing broken doors, providing bins and providing toilet-paper holders.

For peer review only - http://bmjopen.bmj.com/site/about/guidelines.xhtml

Study design and evaluation

The design was a longitudinal cohort study. All participants were asked to self-complete a quantitative survey at baseline (October 2017) and endline (August 2018) on tablets using Open-Data-Kit software. Questions included knowledge of puberty and menstruation, perceptions of menstruation (using a 5-point Likert scale) (Table 1), menstrual management practices at last menstrual period (LMP), reported pain and pain management strategies, leakages during LMP, anxiety, and the 25-item Strengths and Difficulties Questionnaire (SDQ) which is globally used to screen for behavioural markers of potential mental health problems in children and adolescents, covering four domains (hyperactivity, conduct problems, peer problems and emotional problems).[20]

Table 1: Menstruation knowledge, myth and perception questions

Topic	
1. Menstruation knowledge statements (response True/False)	Adolescence is the time between puberty and adulthood
	Changes in the body during puberty happen because of hormones
	The physical changes related to puberty usually start between 10 and 14 years of age in girls, and between 12 and 16 in boys
	Menstrual blood comes from the stomach where food is digested
	Women stop menstruating after the age of about 45-50
	Menstruation in girls and women is normal
	Pregnant women menstruate
	When a girl gets her first period, her body is ready to have children
	During her period a girl can get pregnant
2.Myth statements (response True/False)	Painkillers cause problems having children
	When a girl has her period she is unclean
	Sanitary pads can cause sickness or infection
	It is healthy for a girl to run, dance or cycle during her period
3. Menstrual cycle questions (closed responses)	What is period blood?
	How long does a period usually last?
	How many days are there usually between periods?
4. Menstruation perception statements (response (Yes/No)	I prefer staying at home during my period rather than going to school
	I worry about being teased during my period
	During my period I feel less self-confident than during other days;
	During my period I avoid physical activity (e.g. walking, running)
	I feel anxious about having my next period
	Boys tease me about my period
	Girls tease me about my period
	I feel comfortable to talk to other girls at school about my period
	If I had a problem with managing my period, I would talk to another girl about it

For peer review only - http://bmjopen.bmj.com/site/about/guidelines.xhtml

A nested cohort comprised a random sub-sample of 50 girls per school assessing the feasibility of school attendance and menstruation patterns using self-completed daily diaries. Nested cohort participants were given a booklet each term for daily recording of school and class attendance, menstruation and pain.

Qualitative interviews were used to assess perceptions and acceptability of the intervention, and its perceived impact on school attendance at endline. In-depth interviews (IDIs) were conducted with 20 female students sampled purposively (menstruating S2 girls with different levels of reported baseline school attendance), 10 teachers (six females and four males) and 10 parents who had attended the drama performance. Four focus-group discussions (FGDs) were conducted (two with each sex) at the schools. Semi-structured topic guides were used for both IDIs and FGDs. Interviewers were the same sex as respondents and interviews were digitally recorded with permission.

Research assistants conducted nine unannounced WASH assessment visits per school at baseline and during follow-up, using a standardized checklist to assess availability, accessibility and functioning in terms of sanitary waste disposal, availability of water, soap, hand-washing facilities and toilet paper; cleanliness and privacy.

Finally, a mixed-methods process evaluation was conducted to improve understanding of intervention implementation. Details of this will be reported separately.

Outcomes

Outcomes were knowledge of puberty and menstruation, attitudes towards menstruation and menstrual practices (assessed by the proportion of students answering all knowledge, myth and menstrual cycles correct respectively), knowledge and use of effective pain management methods (assessed by the proportion of girls knowing, and reporting using, at least one

For peer review only - http://bmjopen.bmj.com/site/about/guidelines.xhtml

effective pain relief method during their LMP if they reported pain), psychosocial wellbeing (assessed by the mean SDQ-25 score) and school attendance (assessed by reported days missing school in the daily diaries). The planned sample size of 200 girls (and 200 boys, respectively) provides 85% power to detect an odds ratio of two between baseline and endline assuming baseline prevalence of 20%, allowing for within-individual correlation (intracluster-correlation=0.05).

Data management and analysis

Electronically-captured data were exported to Stata version 15. The paper-based diary data and WASH checklist data were entered using Microsoft Access. For the survey data, the intervention effect on binary outcomes was estimated by comparing endline versus baseline measures, using adjusted odds ratios (AOR) and 95% confidence intervals (CI) estimated with random-effects logistic regression accounting for within-individual clustering. This provides an estimate of the odds ratio for outcomes assessed at endline compared with baseline, independently of potential confounding variables. Effects on continuous outcomes were similarly assessed with random-effects linear regression to estimate the adjusted mean difference (AMD) and 95%CI. All analyses were adjusted for school, gender and age (<16, >16 years) as fixed effects. Effect-moderation by school and age group were assessed using the likelihood ratio test. For the nested cohort, period-days were defined from the diary data as the days of menstruation (bleeding) plus the day prior to menstruation, with sensitivity analyses restricted to days of menstruation only. Audio-recordings were transcribed verbatim and translated into English. Data were analysed using thematic content analysis, with two social scientists independently coding and checking results. Key themes and subthemes related to the objectives of the qualitative research were organised in a matrix and discussed by the team for appropriate interpretation.

For peer review only - http://bmjopen.bmj.com/site/about/guidelines.xhtml

Participant and public involvement

Community groups were involved throughout the formative work, with stakeholders' workshops in August 2016 and October 2018, and a ToC workshop in April 2017. The ToC workshop included 35 invited participants (teachers, students and parents from the MENISCUS-1 and -2 schools, representatives from the Ministry of Education and Sports (MoES), Ministry of Health (MoH), the District Education Officer, Makerere University and NGOs working on menstrual health). In October 2018, we disseminated findings from MENISCUS-2 at a stakeholders' workshop, and elicited input into the future trial design. This meeting was attended by 60 participants, including representatives from MoES, MoH, Uganda National Council for Science and Technology (UNCST), Wakiso District Local Government Makerere University, AFRIpads, WoMena and Entebbe Municipal Council. MENISCUS-1 and two schools were represented by head teachers, students and parents.

RESULTS

Socio-demographic characteristics

Of the 473 eligible S2 students at baseline, 450 (95.1%; 232 girls and 218 boys) consented/assented. Overall, 369 students (188 girls; 81.0%; and 181 boys; 83.0%) participated in the endline survey. At baseline, the mean age was 15.4 years (SD 1.31; range 12-20) for girls and 16.2 (SD=1.5; range 13-21) for boys. Follow-up ranged from 8.6 to 9.6 months (median 9.00 months). Among the girls, 222 (95.7%) and 183 (97.3%) had started menstruating at baseline and endline respectively. The majority (n=361; 80.2%) were Christian and 198 (44.0%) were of Ganda ethnicity. About half (50.6%) of participants' mothers had post-primary education, as did 73.5% of participants' fathers (excluding 74 mothers and 88 fathers where this information was unknown). Relatively few participants (n=59; 13.1%) had running water at home, and 54 (12.0%) had a flush toilet in the home.

For peer review only - http://bmjopen.bmj.com/site/about/guidelines.xhtml

Feasibility and acceptability of implementing the intervention

There were high levels of uptake of the individual and behavioural intervention components (puberty education, drama skit, MHM kit, and pain management). Most students (81.3%) reported attending puberty education sessions and 93.4% of girls reported receiving the MHM kit. Both schools performed the drama skit, and the qualitative interviews indicated that the skit increased MHM awareness and enabled some of the girls to talk about MHM with their parents, especially their fathers.

"When we all saw the skit, it gave us a starting point to initiate a discussion with our children. Even men got it" **(***Female Parent).*

"Trainings improved our self-esteem and confidence because nowadays we are not scared of coming to school. We are comfortable coming to school during our menstruation. Before the training some of us were shy and we couldn't stand and talk in-front of people or parents about menstruation and puberty but now we can," (FGD girls, Private school).

The WASH component was challenging to implement. At baseline, there were no sanitary disposal bins, lockable toilet paper holders, or functional water drums at either school (Table 2). Both schools received these items as part of the intervention. At endline, in the private school, all the girls' cubicles were functional, clean and with a lockable door versus only 33% in the Government school, where facilities were shared with the community. Despite this, most girls (81.4%) reported improvements in toilet facilities at endline, which improved their comfort managing menstruation.

"The school environment is now conducive … during our menstruation because the water is available, they give us water mixed with liquid soap; the toilet doors have locks, there is privacy, a person cannot interrupt you while changing the pad. Toilet paper is available whenever needed and the toilets are always clean." (Female student, Private school).

For peer review only - http://bmjopen.bmj.com/site/about/guidelines.xhtml

Table 2: Summary of WASH facilities at baseline and during implementation

Component	Baseline (1 visit)	Follow up (8 visits)			
	Boys and girls	Girls		Boys	
	Both schools	*Private school*	*Government school*	*Private school*	*Government school*
**Bin**	0%	>75%	100%	n/a	n/a
**Toilet paper**	0%	25%	0%	<25%	<25%
**Functioning water drum**	0%	100%	>75%	100%	>75%
**Functioning water & soap drum**	0%	>75%	25%	50-75%	25-50%

% of the 8 visits when the WASH component was present outside at least one toilet block in the school

For peer review only

Knowledge, misconceptions and perceptions of puberty and menstruation

Knowledge of puberty and menstruation was poor at baseline, especially among boys. The proportion answering all nine knowledge questions correctly increased from 11.6% to 23.9% (AOR=4.03, 95%CI 1.88-8.68) in girls, and from 4.1% to 12.7% (AOR=4.11, 95%CI 1.62-10.5) in boys (Table 3). There was also evidence of improvements in knowledge of the menstrual cycle, particularly among girls, although endline knowledge remained poor (29.3% of girls and 7.7% of boys answering all 3 questions correctly; Table 3).

The proportion of girls answering at least eight of nine questions on their perceptions about menstruation positively (i.e. questions about self-confidence, teasing and anxiety) increased from 12.2% at baseline to 27.9% at endline (AOR=3.62, 95%CI: 1.85-7.11). The largest effects were reductions in the proportion of girls reporting teasing by boys about menstruation (14.4% to 8.7%, AOR=0.43, 95%CI 0.19-0.96), girls feeling anxious about the next period (58.6% to 34.4%, AOR=0.14, 95%CI 0.06-0.31) and girls avoiding physical activity during menstruation (47.8% to 25.7%; AOR=0.27, 95%CI 0.15-0.50). There was no evidence of moderation by age (Table 4; p≥0.2 for effect modification for each variable) or school (p≥0.1 for effect modification for each variable; results not shown).

Table 3: Knowledge of puberty and menstruation, attitudes to menstruation and menstrual practices at baseline and endline, by gender

	Girls			Boys		
	**Baseline (n=232)**	**Endline (n=188)**	**AOR (95% CI)**	**Baseline (n=218)**	**Endline (n=181)**	**AOR (95% CI)**
All 9 knowledge questions correct[1]	27 (11.6%)	45 (23.9%)	4.03 (1.88-8.68)	9 (4.1%)	23 (12.7%)	4.11 (1.62-10.5)
Knowledge excluding fertility question	70 (30.2%)	120 (63.8%)	7.02 (3.67-13.45)	42 (19.3%)	73 (40.3%)	4.16 (2.15-8.04)
All 4 myth questions correct[2]	39 (16.8%)	77 (41.0%)	5.44 (2.82-10.50)	22 (10.1%)	44 (24.3%)	3.99 (2.00-8.04)
All 3 menstrual cycle questions[3]	12 (5.2%)	55 (29.3%)	19.45 (5.88-64.5)	4 (1.8%)	14 (7.7%)	3.95 (1.26-12.4)

[1] Knowledge statements were: i) Adolescence is the time between puberty and adulthood; ii) Changes in the body during puberty happen because of hormones; iii) The physical changes related to puberty usually start between 10 and 14 years of age in girls, and between 12 and 16 in boys; iv) Menstrual blood comes from the stomach where food is digested; v) Women stop menstruating after the age of about 45-50; vi) Menstruation in girls and women is normal; vii) Pregnant women menstruate; viii) When a girl gets her first period, her body is ready to have children; ix) During her period a girl can get pregnant
[2] Myth statements were: i) Painkillers cause problems having children; ii) When a girl has her period she is unclean; iii) Sanitary pads can cause sickness or infection; iv) It is healthy for a girl to run, dance or cycle during her period
[3] Menstrual cycle questions were: i) What is period blood?; ii) How long does a period usually last?; iii) How many days are there usually between periods?

For peer review only - http://bmjopen.bmj.com/site/about/guidelines.xhtml

In qualitative interviews, girls and teachers said that they thought that fear, myths and negative perceptions about menstruation were due to lack of knowledge. Participants suggested that the intervention improved knowledge about puberty, menstruation, and girls' accounts suggested a reduction of teasing by boys at school:

"Before MENISCUS, boys used to laugh at girls, for example when a girl stood up in class with her dress stained, boys would laugh at her but after MENISCUS training, they stopped laughing at girls and they now care about us" (Female student, Private school).

Management of menstruation

The proportion of girls reporting using manufactured menstrual materials exclusively (i.e. reusable or disposable pads, tampons or menstrual cups) during their LMP increased from 73.0% at baseline to 89.1% at endline (AOR=4.14, 95%CI 2.04-8.40). At endline, most girls (n=155; 82.5%) reported using reusable pads during their LMP compared with 18.5% at baseline. There was some evidence of a decrease in reported leakage of blood through their clothing during LMP (47.4% at baseline vs 35.3% at endline; AOR=0.51, 95%CI 0.25-1.04), but no evidence of a difference in the proportion of girls reporting staining their underwear during their LMP (23.4% at baseline to 27.3% at endline (AOR=1.34, 95%CI 0.78-2.30). There was no evidence of effect-moderation by age or school (Table 4).

Knowledge and use of effective pain management methods

Most girls reported pain during menstruation at both baseline and endline (74.3% and 71.0%, respectively). Amongst these girls, there was evidence of an increase in the proportion who reported using painkillers during their LMP (46.7% at baseline to 60.8% at endline; AOR=1.87, 95%CI 1.05-3.34). Results were similar by school and age (Table 4). There was evidence of an increase in the proportion of girls who reported using ≥1 effective pain relief method during their LMP (76.4% at baseline to 91.5% at endline; AOR=3.43, 95%CI 1.58-7.40). Of the 232

For peer review only - http://bmjopen.bmj.com/site/about/guidelines.xhtml

girls who received a voucher for painkillers, 58 (25.0%) had redeemed 78 vouchers by endline: 77% used one voucher, 16% used two, and 7% used $\geq$2.

The qualitative findings confirmed that non-pharmacological methods of pain relief were popular and effective.

"They [MENISCUS team] taught us how to do exercises to relieve pain and it worked for me so, the last time, I didn't use painkillers; I managed my periods by doing exercises and using reusable pads MENISCUS had provided." (Female student, Government school).

Behavioural markers of psychosocial well-being

The mean SDQ-25 score in girls decreased from 10.3 to 9.2 (p=0.006, adjusted for age and school), indicating improved behaviour and conduct. No decrease was seen among boys, in line with the ToC (9.88 at baseline vs 9.91 at endline; p=0.98).

Menstruation and school absence

In the cross-sectional surveys, a similar proportion of girls reported missing at least one school day due to menstruation in Term 2, 2017 (pre-baseline) and Term 2, 2018 (endline) (32.0% vs 32.8%; OR=0.97, 95%CI 0.57-1.66). Among the 100 cohort participants, data were collected from 81 students at endline. The AOR associated with missing school on period-days compared to non-period days decreased from OR=2.17 (95%CI 1.61-2.92) at baseline to OR=1.29 (95%CI 1.02-1.63) at endline (p-value for moderation =0.01; Table 5). Results were similar for class attendance (Table 4). The direct observation checks found high correlation with the self-completed diaries. Girls were seen on 328/330 (99.4%) of days when their diary stated they were present and were not seen on all 37 days when the diary stated that they were absent. School registers were incomplete and rarely completed by teachers.

For peer review only - http://bmjopen.bmj.com/site/about/guidelines.xhtml

Table 4: Reported perceptions of menstruation, menstrual management, pain and pain management among girls, by age at baseline

	Age <16 years			Age ≥ 16 years		
	**Baseline (n=115)**	**Endline (n=51)**	**AOR (95% CI)**	**Baseline (n=107)**	**Endline (n=132)**	**AOR (95% CI)**
≥8 of 9 positive perceptions of menstruation[1]	14 (12.2%)	16 (31.4%)	5.25 (1.10-25.19)	13 (12.2%)	35 (26.5%)	2.98 (1.29-6.86)
Used manufactured menstrual materials only at LMP	86 (74.8%)	46 (90.2%)	3.40 (1.06-10.9)	76 (71.0%)	117 (88.6%)	4.24 (1.68-10.8)
Leaked blood at LMP	37 (50.0%)	12 (42.9%)	0.60 (0.18-2.04)	37 (45.1%)	24 (32.4%)	0.58 (0.28-1.18)
Underwear stained at LMP	31 (27.0%)	16 (31.4%)	1.14 (0.45-2.87)	21 (19.6%)	34 (25.8%)	1.49 (0.73-3.06)
Knew ≥4 effective pain management methods	30 (24.2%)	37 (67.3%)	10.80 (3.48-33.6)	30 (27.8%)	97 (72.9%)	21.62 (5.44-84.9)
Reported pain at last period	79 (68.7%)	36 (70.6%)	1.00 (0.37-2.65)	86 (80.4%)	94 (71.2%)	0.54 (0.26-1.10)
Used ≥1 effective pain management method[2]	57 (72.2%)	34 (94.4%)	6.78 (1.49-30.8)	69 (80.2%)	85 (90.4%)	2.79 (0.95-8.21)
Used painkillers at LMP[3]	32 (40.5%)	21 (58.3%)	2.16 (0.91-5.11)	45 (52.3%)	58 (61.7%)	1.70 (0.74-3.92)
Used other effective methods[2]	39 (49.4%)	29 (80.6%)	4.22 (1.66-10.78)	43 (50.0%)	72 (76.6%)	6.83 (1.88-24.9)
Did nothing for pain at LMP[1]	20 (25.3%)	2 (5.6%)	0.17 (0.04-0.79)	17 (19.8%)	7 (7.5%)	0.18 (0.03-0.99)

For peer review only

[1] Perception questions were i) I prefer staying at home during my period rather than going to school; ii) I worry about being teased during my period; iii) During my period I feel less self-confident than during other days; iv) During my period I avoid physical activity (e.g. walking, running); v) I feel anxious about having my next period; vi) Boys tease me about my period vii) Girls tease me about my period; viii) I feel comfortable to talk to other girls at school about my period; ix) If I had a problem with managing my period, I would talk to another girl about it

[2] Effective methods (painkiller, drinking water, using hot water bottle, exercise, relaxing, foods with lots of water).

[3] Among those with pain at LMP

For peer review only - http://bmjopen.bmj.com/site/about/guidelines.xhtml

Table 5: School and class attendance at baseline and endline among nested cohort participants, by period-day status

	Term 3 2017 (baseline) 99 girls		Term 2 2018 (end-line) 81 girls	
	**Non period-day**	**Period-day[1]**	**Non period-day**	**Period-day**
Number of days	2625	554	3829	787
N (%) not attending full day of school	8.5%	14.6%	11.6%	14.2%
**Odds ratio (95% CI)[2]**	OR=2.17 (1.61-2.92)		OR=1.29 (1.02-1.63)	
N (%) not attending all classes	11.4%	19.9%	17.3%	20.5%
Odds ratio (95% CI)	OR=2.12 (1.64-2.74)		OR=1.26 (1.04-1.54)	

The qualitative interviews suggested that girls were more likely to attend school during menstruation at endline than at baseline, with reasons including the training on pain management, tracking their menstrual cycle, and having re-usable pads.

"Some girls totally don't come to school every menstruation period. However, in our class, missing school is now rare because we have learnt how to manage the periods, pain and we have what to use. We now also know our cycle" (Girls FGD; Government school).

"Before MENISCUS study gave us diaries, I didn't know how to track my days. I used to come to school and my uniform would get soiled. Sometimes, I could ask for a pass out to go back home during my periods. […] But when MENISCUS came, they gave us diaries, I started filling them in and now I know my days; I come to school prepared." (Female, Private school).

[1] Includes days of menses plus day prior to menses (results similar when restricted to days of menses) obtained from daily diaries for 9 months
[2] Obtained from random effects logistic regression, allowing for within-girl clustering

For peer review only - http://bmjopen.bmj.com/site/about/guidelines.xhtml

DISCUSSION

To our knowledge, this is the first MHM intervention to be pilot tested that focused equally on health promotion (psychosocial issues, stigma and knowledge) and goods (provision of pads and analgesics, and improved WASH facilities).[21]

Feasibility and accessibility of implementing the intervention

The qualitative and quantitative data showed that the intervention was feasible and acceptable to schools and stakeholders. A detailed process evaluation will be published separately. The main challenge with implementation was maintenance of the WASH component. To improve this component in a future trial, we will ask schools to establish an MHM Leadership Group (to include teachers, parents and students) responsible for ensuring each intervention element is delivered, maintained and sustained by the schools. Another challenge was that we did not obtain ethical approval to offer girls a menstrual cup, due to concerns about whether cup insertion could lead to damage of the hymen and affect a girl's virginity. Further activities to inform parents about menstrual cups and discuss concerns are underway.

Potential intervention effect on knowledge, menstrual practices and school attendance

The study was designed to assess feasibility and accessibility of implementing the intervention, rather than assess the effectiveness. Although the findings suggest a potential intervention effect on education and health outcomes, these results need to be interpreted cautiously as the improvements reported may be partly due to i) older age at endline – there may have been improvements in knowledge and self-confidence in managing menstruation due to a cohort effect; ii) reporting biases - the outcome assessment team were involved with implementation and this may have caused social desirability bias affecting responses on attitudes or behaviours following the intervention; iii) biases due to the lack of a comparison group, and the endline survey being conducted in a different term to baseline. For example,

For peer review only - http://bmjopen.bmj.com/site/about/guidelines.xhtml

girls reported higher levels of school attendance overall at baseline than at endline, and this may be due to the baseline being conducted during an examination term. Iv) generalisability – the extent to which are findings will hold in other geographic or socio-economic settings is not clear.

The study highlighted poor knowledge of menstruation, possibly due to cultural taboos and norms [16, 22], and lack of knowledge among students [23] parents and teachers hindering discussion of puberty and menstruation.[16, 24-26] Three intervention components addressed knowledge (puberty education, menstrual-management kit training, and drama skit). To address stigma and effect a school-wide change in attitudes, the education and drama sessions included boys. The qualitative results indicated a preference for a boys-only puberty education session, and this would be included in a future intervention. This pilot study showed that girls were less anxious about menstruation, and reported less teasing by boys after the intervention, supporting the theory of change, and previous qualitative work from Ghana that indicated reductions in shame and improvements in self-confidence following provision of pads and puberty education.[27] It also worth noting that although the SDQ has proven reliability and validity in a number of studies across Europe, Asia, Australia and South America[28-30], and has been used widely in Africa,[31] there has only been one psychometric validation of the tool which found satisfactory internal consistency.[32]

At endline, compared with baseline, more girls reported use of only manufactured materials during their LMP, and using these correctly. The high uptake of reusable pads was expected, as these were provided free to all girls, but the continued use over follow-up, and favourable qualitative reports, shows that pads were acceptable. Similar findings on correct use have been seen in another study in Uganda where 98% of girls reported washing and using soap for their AFRIpads [21] and in India,[33] where a school-based health-education intervention led to improvements in washing clothes with soap, drying them in the sun and safe disposal.

For peer review only - http://bmjopen.bmj.com/site/about/guidelines.xhtml

Previous studies show inconsistent evidence for a relationship between menstruation and school absenteeism.[15, 16, 34-41] Documenting school attendance is often challenging in LMICs due to incomplete or inaccurate school registers.[7, 16] Our diaries were popular and correlated well with direct observation. The improved school attendance during menstruation in this study was attributed to improved pain management, knowledge to track menstrual cycles, and the provision of reusable pads in qualitative interviews.

Innovative aspects of the MENISCUS intervention

1) Multi-component focus. Past and ongoing studies tend to focus on single-component interventions.[7, 37] This study indicates synergies between the intervention elements e.g. the drama skit reinforces puberty knowledge, reduces stigma and engages parents and boys. An emerging view, aligned with our findings, is that poor MHH is a social problem and to be effective, MHH interventions in many settings should address the broader issues of menstrual stigma and literacy as well as the provision of menstrual products or improving WASH facilities.[9] The multi-component approach addresses both individual, behavioural and environmental barriers to good MHH and school attendance.

2) Pain management. Menstrual pain is a major contributor to school absence in girls in many settings.[16, 22, 42, 43] This is the first MHH intervention to address this issue and we found substantial improvements in pain management.[16] Further research is needed on this topic as misconceptions about use of painkillers are common in Uganda [16] and elsewhere.[44, 45]

3) Inclusion of boys. Most MHH interventions focus only on girls, yet sustainable changes in MHM depend on addressing stigma about menstruation. This study highlighted the importance of including boys which is aligned with SCT (i.e. the intervention provides positive reinforcement for behavioural change by improving the school environment). The focus on boys as well as girls is supported by WHO framework for Health Promoting Schools [46] and

For peer review only - http://bmjopen.bmj.com/site/about/guidelines.xhtml

is aligned with a report commissioned by the Gates Foundation on Menstrual Health and Gender Equity.[47]

4) Urban secondary school setting. Most intervention studies to date have been in primary schools, and in rural areas.[21, 42] MHH and school attendance is an issue in the peri-urban area in Entebbe where most girls can afford to buy disposable pads for some of the time.[16] A focus on MHH in secondary schools in different settings is important given the recognised importance of girls' secondary education to future development,[48] and evidence that school absenteeism due to menstruation is problematic in secondary school girls.[16, 49]

CONCLUSION

This pilot study showed that the MHH intervention was acceptable and feasible to implement. There were substantial reported improvements in stigma and anxiety, pain management, and some improvement in MHH knowledge of puberty and menstruation and WASH following a multi-component intervention. Based on SCT, we anticipate the results to be generalizable to other similar contexts with relatively low levels of MHM knowledge, stigma about discussing menstruation, and poor school WASH facilities. A phase-III trial is warranted to evaluate the impact of the intervention on school attendance, and on health, well-being and educational outcomes for definitive results to drive forward policy changes.

For peer review only

REFERENCES

1. Das P, Baker KK, Dutta A, et al. Menstrual Hygiene Practices, WASH Access and the Risk of Urogenital Infection in Women from Odisha, India. PLoS One. 2015;10(6):e0130777.
2. Sommer M, Caruso BA, Sahin M, et al. A Time for Global Action: Addressing Girls' Menstrual Hygiene Management Needs in Schools. PLoS Med. 2016;13(2):e1001962.
3. Sommer M, Sahin M. Overcoming the taboo: advancing the global agenda for menstrual hygiene management for schoolgirls. Am J Public Health. 2013;103(9):1556-9.
4. Sommer M, Hirsch JS, Nathanson C, et al. Comfortably, Safely, and Without Shame: Defining Menstrual Hygiene Management as a Public Health Issue. Am J Public Health. 2015;105(7):1302-11.
5. UNICEF. Guidance on menstrual health and hygiene. New York: UNICEF, 2019.
6. Hennegan J, Shannon AK, Rubli J, et al. Women's and girls' experiences of menstruation in low- and middle-income countries: A systematic review and qualitative metasynthesis. PLoS Med. 2019;16(5):e1002803.
7. Sumpter C, Torondel B. A systematic review of the health and social effects of menstrual hygiene management. PLoS One. 2013;8(4):e62004.
8. Elledge MF, Muralidharan A, Parker A, et al. Menstrual Hygiene Management and Waste Disposal in Low and Middle Income Countries-A Review of the Literature. 2018;15(11).
9. Bobel C. The Managed Body: Developing Girls and Menstrual Health in the Global South: Palgrave Macmillan; 2018.
10. Hennegan J. Menstrual Hygiene Management and Human Rights: The Case for an Evidence-Based Approach. Women's Reproductive Health. 2017;4(3):212-31.
11. Phillips-Howard PA, Caruso B, Torondel B, et al. Menstrual hygiene management among adolescent schoolgirls in low- and middle-income countries: research priorities. Glob Health Action. 2016;9:33032.
12. United Nations International Children's Emergency Fund, World Health Organisation. Drinking Water, Sanitation and Hygiene in Schools: Global baseline report 2018. New York: . 2018.
13. United Nations International Children's Emergency Fund. MHM in Ten; Advancing the MHM agenda in schools. Third annual meeting co-hosted by Columbia University and UNICEF New York. http://www.ungei.org/2016_MHM_in_Ten_Meeting_Report.pdf. 2016.
14. UNICEF. MHM in Ten: Advancing the MHM agenda in WASH in Schools. New York2014.
15. Hennegan J, Montgomery P. Do Menstrual Hygiene Management Interventions Improve Education and Psychosocial Outcomes for Women and Girls in Low and Middle Income Countries? A Systematic Review. PLoS One. 2016;11(2):e0146985.
16. Miiro G, Rutakumwa R, Nakiyingi-Miiro J, et al. Menstrual health and school absenteeism among adolescent girls in Uganda (MENISCUS): a feasibility study. BMC Womens Health. 2018;18(1):4.
17. MoEST. Menstruation: Breaking the silence, taking action. Kampala: 2015.
18. Ali CS. Provision of menstrual hygiene management facilities for girls and female teachers in primary and secondary schools in Uganda : study on implementation of the Ministry of Education and Sports circular. Kampala, Uganda: IRC Wash, 2018 Contract No.: 84572.
19. Bandura A. Health promotion by social cognitive means. Health Educ Behav. 2004;31(2):143-64.
20. Deighton J, Croudace T, Fonagy P, et al. Measuring mental health and wellbeing outcomes for children and adolescents to inform practice and policy: a review of child self-report measures. Child Adolesc Psychiatry Ment Health. 2014;8:14.

For peer review only - http://bmjopen.bmj.com/site/about/guidelines.xhtml

21. Hennegan J, Dolan C, Wu M, et al. Schoolgirls' experience and appraisal of menstrual absorbents in rural Uganda: a cross-sectional evaluation of reusable sanitary pads. Reprod Health. 2016;13(1):143.
22. UNICEF Western and Central A firica Regional Office. Menstrual Hygiene in Schools in 2 countries of Francophone West Africa; Burkina Faso and Niger Case Studies. 2013.
23. Shanbhag D, Shilpa R, D'Souza N, et al. Perceptions regarding menstruation and Practices during menstrual cycles among high school going adolescent girls in resource limited settings around Bangalore City, Karnataka, India. . Int J Collab Res Inter Med Public Health. 2012;4(7):1353–62.
24. Chandra-Mouli V, Patel SV. Mapping the knowledge and understanding of menarche, menstrual hygiene and menstrual health among adolescent girls in low- and middle-income countries. Reprod Health. 2017;14(1):30.
25. McMahon SA, Winch PJ, Caruso BA, et al. 'The girl with her period is the one to hang her head' Reflections on menstrual management among schoolgirls in rural Kenya. BMC Int Health Hum Rights. 2011;11:7.
26. Bosch AM, Hutter I, van Ginneken JK. Perceptions of adolescents and their mothers on reproductive and sexual development in Matlab, Bangladesh. Int J Adolesc Med Health. 2008;20(3):329-42.
27. Dolan CS, Ryus CR, Dopson S, et al. A blind spot in girls' education: menarche and its webs of exclusion in Ghana. Journal of International Development. 2013;26(5):643±57.
28. Woerner W, Fleitlich-Bilyk B, Martinussen R, et al. The Strengths and Difficulties Questionnaire overseas: evaluations and applications of the SDQ beyond Europe. Eur Child Adolesc Psychiatry. 2004;13 Suppl 2:II47-54.
29. Vostanis P. Strengths and Difficulties Questionnaire: research and clinical applications. Curr Opin Psychiatry. 2006;19(4):367-72.
30. Goodman R. The Strengths and Difficulties Questionnaire: a research note. J Child Psychol Psychiatry. 1997;38(5):581-6.
31. Hoosen N, Davids EL, de Vries PJ, et al. The Strengths and Difficulties Questionnaire (SDQ) in Africa: a scoping review of its application and validation. Child Adolesc Psychiatry Ment Health. 2018;12:6.
32. Kashala E, Elgen I, Sommerfelt K, et al. Teacher ratings of mental health among school children in Kinshasa, Democratic Republic of Congo. Eur Child Adolesc Psychiatry. 2005;14(4):208-15.
33. Nemade D, Anjenaya S, Gujar R. Impact of health education on knowledge and practices about menstruation among adolescent school girls of Kalamboli, Navi-Mumbai. . Health Popul Perspect Issues. 2009;32(4):167–75.
34. Grant MJ, Lloyd CB, Mensch BS. Menstruation and School Absenteeism: Evidence from Rural Malawi. Comp Educ Rev. 2013;57(2):260-84.
35. van Eijk AM, Sivakami M, Thakkar MB, et al. Menstrual hygiene management among adolescent girls in India: a systematic review and meta-analysis. BMJ Open. 2016;6(3):e010290.
36. Oster E, Thornton R. Menstruation, Sanitary Products, and School Attendance: Evidence from a Randomized Evaluation. American Economic Journal: Applied Economics. 2011;3(1):91-100.
37. Phillips-Howard PA, Nyothach E, Ter Kuile FO, et al. Menstrual cups and sanitary pads to reduce school attrition, and sexually transmitted and reproductive tract infections: a cluster randomised controlled feasibility study in rural Western Kenya. BMJ Open. 2016;6(11):e013229.
38. Tegegne TK, Sisay MM. Menstrual hygiene management and school absenteeism among female adolescent students in Northeast Ethiopia. BMC Public Health. 2014;14:1118.
39. Montgomery P, Ryus CR, Dolan CS, et al. Sanitary pad interventions for girls' education in Ghana: a pilot study. PLoS One. 2012;7(10):e48274.
40. Wilson E, Reeve J, Pitt A. Education. Period. Developing an acceptable and replicable menstrual hygiene intervention. Development in Practice 2014;24(1):63±80.

For peer review only - http://bmjopen.bmj.com/site/about/guidelines.xhtml

41. Scott L, Dopson S, Montgomery P, et al. Impact of Providing Sanitary Pads To Poor Girls in Africa. University of Oxford. 2009.
42. Boosey R, Prestwich G, Deave T. Menstrual hygiene management amongst schoolgirls in the Rukungiri district of Uganda and the impact on their education: a cross-sectional study. Pan Afr Med J. 2014;19:253.
43. Crofts T, J. F. Menstrual hygiene in Ugandan schools: an investigation of low-cost sanitary pads. J Water Sanitation Hyg Dev. 2012;2:50–8.
44. Kontogiorgis C, Nena E, Berberoglou E, et al. Estimating Consumers' Knowledge and Attitudes Towards Over-The-Counter Analgesic Medication in Greece in the Years of Financial Crisis: The Case of Paracetamol. Pain Ther. 2016;5(1):19-28.
45. Palos GR, Mendoza TR, Cantor SB, et al. Perceptions of analgesic use and side effects: what the public values in pain management. J Pain Symptom Manage. 2004;28(5):460-73.
46. Langford R, Bonell C, Jones H, et al. The World Health Organization's Health Promoting Schools framework: a Cochrane systematic review and meta-analysis. BMC Public Health. 2015;15:130.
47. Geertz A, Iyer L, Kasen P, et al. An opportunity to address menstrual health and gender equity. Bill & Melinda Gates Foundation, May 2016.
48. Wodon QT, Montenegro CE, Nguyen H, et al. Missed Opportunities : The High Cost of Not Educating Girls (English). The Cost of Not Educating Girls Series. Washington, D.C. : World Bank Group. http://documents.worldbank.org/curated/en/775261531234655903/Missed-Opportunities-The-High-Cost-of-Not-Educating-Girls. 2018.
49. Vashisht A, Pathak R, Agarwalla R, et al. School absenteeism during menstruation amongst adolescent girls in Delhi, India. J Family Community Med. 2018;25(3):163-8.

Figure Legend:

Figure 1: Theory of Change for the MENISCUS intervention

Statements:

Contributors: HAW, LH, BT, DAR, JS, CB, SNe, SCF and CA contributed to the overall conception and design of the study. CK, LH, SNa, RN, KN, PN, SNM undertook the data collection. CK and HAW wrote the first draft of the manuscript. All authors contributed to the interpretation of results and drafting of the manuscript. HAW and CT did the statistical analyses. All authors take responsibility for the integrity of the data and the accuracy of the data analysis. All authors read and approved the final manuscript. HAW is the guarantor.

Funding statement: This work was supported by a United Kingdom (UK) Department for International Development (DFID), the National Institute for Health Research (NIHR), UK Medical Research Council (MRC), and Wellcome Trust Joint Global Health Trials

For peer review only - http://bmjopen.bmj.com/site/about/guidelines.xhtml

Development Grant (number MR/P020283/1). The authors would like to thank the study participants, contact teachers in the two schools, and the Ministry of Education and Sports and Ministry of Health Uganda for their support.

Competing Interests: None declared

Disclaimer: The content is solely the responsibility of the authors and does not necessarily represent the official views of the funders.

Ethical approval: Ethics approval was received from the UVRI Research Ethics Committee, the Uganda National Council for Science and Technology (UNCST) and the London School of Hygiene and Tropical Medicine.

Data sharing statement: Data will be made available in the LSHTM Data Compass repository on request from the corresponding author (Helen Weiss https://orcid.org/0000-0003-3547-7936) from the website https://datacompass.lshtm.ac.uk/

Patient request for publication: Not required

For peer review only

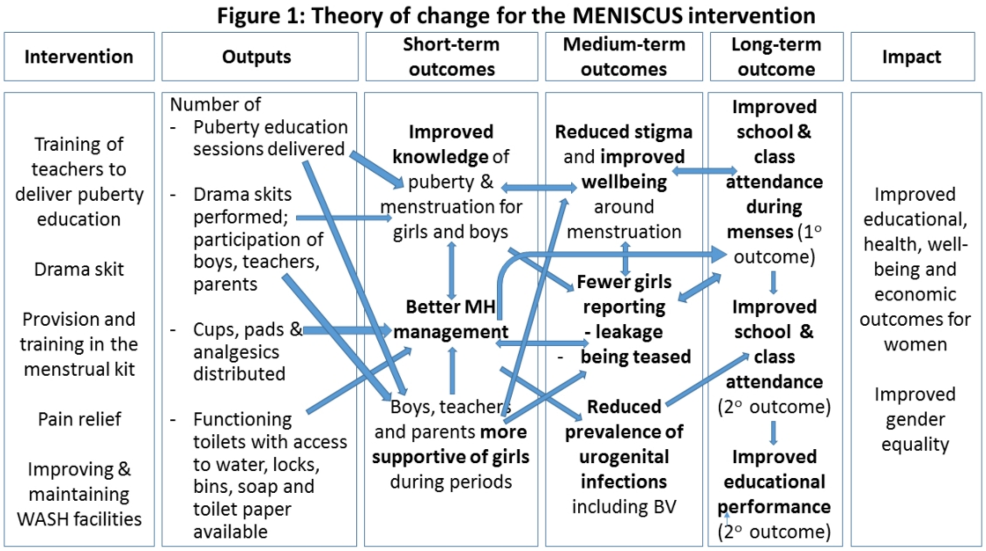

Figure 1: Theory of Change for MENISCUS intervention

338x190mm (96 x 96 DPI)

STROBE Statement—Checklist of items that should be included in reports of ***cohort studies***

	Item No	Recommendation	Page number
**Title and abstract**	1	(*a*) Indicate the study's design with a commonly used term in the title or the abstract	1
		(*b*) Provide in the abstract an informative and balanced summary of what was done and what was found	2
**Introduction**			
Background/rationale	2	Explain the scientific background and rationale for the investigation being reported	4-5
Objectives	3	State specific objectives, including any prespecified hypotheses	5
**Methods**			
Study design	4	Present key elements of study design early in the paper	8
Setting	5	Describe the setting, locations, and relevant dates, including periods of recruitment, exposure, follow-up, and data collection	8,11
Participants	6	(*a*) Give the eligibility criteria, and the sources and methods of selection of participants. Describe methods of follow-up	8,11
		(*b*) For matched studies, give matching criteria and number of exposed and unexposed	n/a
Variables	7	Clearly define all outcomes, exposures, predictors, potential confounders, and effect modifiers. Give diagnostic criteria, if applicable	6, 9
Data sources/ measurement	8*	For each variable of interest, give sources of data and details of methods of assessment (measurement). Describe comparability of assessment methods if there is more than one group	8,9
Bias	9	Describe any efforts to address potential sources of bias	10
Study size	10	Explain how the study size was arrived at	10
Quantitative variables	11	Explain how quantitative variables were handled in the analyses. If applicable, describe which groupings were chosen and why	10
Statistical methods	12	(*a*) Describe all statistical methods, including those used to control for confounding	10
		(*b*) Describe any methods used to examine subgroups and interactions	10
		(*c*) Explain how missing data were addressed	n/a
		(*d*) If applicable, explain how loss to follow-up was addressed	n/a
		(*e*) Describe any sensitivity analyses	n/a
**Results**			
Participants	13*	(a) Report numbers of individuals at each stage of study—eg numbers potentially eligible, examined for eligibility, confirmed eligible, included in the study, completing follow-up, and analysed	11
		(b) Give reasons for non-participation at each stage	n/a
		(c) Consider use of a flow diagram	n/a
Descriptive data	14*	(a) Give characteristics of study participants (eg demographic, clinical, social) and information on exposures and potential confounders	11
		(b) Indicate number of participants with missing data for each variable of interest	11
		(c) Summarise follow-up time (eg, average and total amount)	11
Outcome data	15*	Report numbers of outcome events or summary measures over time	13-17
Main results	16	(*a*) Give unadjusted estimates and, if applicable, confounder-adjusted estimates and their precision (eg, 95% confidence interval). Make clear	13-17

For peer review only

For peer review only - http://bmjopen.bmj.com/site/about/guidelines.xhtml

		which confounders were adjusted for and why they were included	
		(*b*) Report category boundaries when continuous variables were categorized	13-17
		(*c*) If relevant, consider translating estimates of relative risk into absolute risk for a meaningful time period	n/a
Other analyses	17	Report other analyses done—eg analyses of subgroups and interactions, and sensitivity analyses	13 and 15
**Discussion**			
Key results	18	Summarise key results with reference to study objectives	19
Limitations	19	Discuss limitations of the study, taking into account sources of potential bias or imprecision. Discuss both direction and magnitude of any potential bias	19
Interpretation	20	Give a cautious overall interpretation of results considering objectives, limitations, multiplicity of analyses, results from similar studies, and other relevant evidence	20-21
Generalisability	21	Discuss the generalisability (external validity) of the study results	21
**Other information**			
Funding	22	Give the source of funding and the role of the funders for the present study and, if applicable, for the original study on which the present article is based	25

*Give information separately for exposed and unexposed groups.

Note: An Explanation and Elaboration article discusses each checklist item and gives methodological background and published examples of transparent reporting. The STROBE checklist is best used in conjunction with this article (freely available on the Web sites of PLoS Medicine at http://www.plosmedicine.org/, Annals of Internal Medicine at http://www.annals.org/, and Epidemiology at http://www.epidem.com/). Information on the STROBE Initiative is available at http://www.strobe-statement.org.

For peer review only

BMJ Open

Menstrual health intervention and school attendance in Uganda (MENISCUS-2): A pilot intervention study

Journal:	*BMJ Open*
Manuscript ID	bmjopen-2019-031182.R2
Article Type:	Original research
Date Submitted by the Author:	05-Dec-2019
Complete List of Authors:	Kansiime, Catherine; Medical Research Council / Uganda Virus Research Institute & London School of Hygiene & Tropical Medicine Uganda Research Unit<br>Hytti, Laura; WoMena Uganda<br>Nalugya, Ruth; Medical Research Council / Uganda Virus Research Institute & London School of Hygiene & Tropical Medicine Uganda Research Unit<br>Nakuya, Kevin; Medical Research Council / Uganda Virus Research Institute & London School of Hygiene & Tropical Medicine Uganda Research Unit<br>Namirembe, Prossy; Medical Research Council / Uganda Virus Research Institute & London School of Hygiene & Tropical Medicine Uganda Research Unit<br>Nakalema, Shamirah; WoMena Uganda<br>Neema, Stella; Makerere University, College of Humanities and Social Science<br>Tanton, Clare; London School of Hygiene and Tropical Medicine, Epidemiology and Population Health<br>Alezuyo, Connie; Palm Tree Academy Uganda<br>Namuli Musoke, Saidat; Uganda Virus Research Institute<br>Torondel, Belen; London School of Hygiene and Tropical Medicine, Faculty of Infectious and Tropical Diseases<br>Francis, Suzanna ; London School of Hygiene and Tropical Medicine, MRC Tropical Epidemiology Group, Faculty of Epidemiology and Population Health<br>Ross, David; World Health Organization, Department of Maternal, Newborn, Child and Adolescent Health<br>Bonell, Christopher; London School of Hygiene and Tropical Medicine, Public Health and Policy<br>Seeley, Janet; London School of Hygiene and Tropical Medicine Faculty of Public Health and Policy; Medical Research Council / Uganda Virus Research Institute & London School of Hygiene & Tropical Medicine Uganda Research Unit<br>Weiss, Helen; London School of Hygiene and Tropical Medicine, MRC Tropical Epidemiology Group, Faculty of Epidemiology and Population Health
**Primary Subject Heading**:	Global health
Secondary Subject Heading:	Epidemiology

Keywords:	Menstrual Health, School attendance, Menstrual Hygiene, Adolescent Health, Education

SCHOLARONE™
Manuscripts

I, the Submitting Author has the right to grant and does grant on behalf of all authors of the Work (as defined in the below author licence), an exclusive licence and/or a non-exclusive licence for contributions from authors who are: i) UK Crown employees; ii) where BMJ has agreed a CC-BY licence shall apply, and/or iii) in accordance with the terms applicable for US Federal Government officers or employees acting as part of their official duties; on a worldwide, perpetual, irrevocable, royalty-free basis to BMJ Publishing Group Ltd ("BMJ") its licensees and where the relevant Journal is co-owned by BMJ to the co-owners of the Journal, to publish the Work in this journal and any other BMJ products and to exploit all rights, as set out in our licence.

The Submitting Author accepts and understands that any supply made under these terms is made by BMJ to the Submitting Author unless you are acting as an employee on behalf of your employer or a postgraduate student of an affiliated institution which is paying any applicable article publishing charge ("APC") for Open Access articles. Where the Submitting Author wishes to make the Work available on an Open Access basis (and intends to pay the relevant APC), the terms of reuse of such Open Access shall be governed by a Creative Commons licence – details of these licences and which Creative Commons licence will apply to this Work are set out in our licence referred to above.

Other than as permitted in any relevant BMJ Author's Self Archiving Policies, I confirm this Work has not been accepted for publication elsewhere, is not being considered for publication elsewhere and does not duplicate material already published. I confirm all authors consent to publication of this Work and authorise the granting of this licence.

For peer review only

Menstrual health intervention and school attendance in Uganda (MENISCUS-2): A pilot intervention study

Catherine Kansiime, Ph.D[a], Laura Hytti, MMSc[b], Ruth Nalugya[a], Kevin Nakuya[a], Prossy Namirembe[a], Shamirah Nakalema[b], Stella Neema, Ph.D[d], Clare Tanton, Ph.D[c], Connie Alezuyo[e], Saidat Namuli Musoke[f], Belen Torondel, Ph.D[g], Suzanna C. Francis, Ph.D[c], David A Ross, Ph.D[h], Christopher Bonell, Ph.D[c], Janet Seeley, Ph.D[a,c], *Helen A. Weiss, Ph.D[c,h].

[a] Medical Research Council / Uganda Virus Research Institute & London School of Hygiene & Tropical Medicine Uganda Research Unit, Plot 51-59 Nakiwogo Road Entebbe, Uganda, [b]WoMena Uganda, Plot 2150 Kaduyu Close, Ntinda-Kigowa Kampala, Uganda, [c] MRC Tropical Epidemiology Group, London School of Hygiene & Tropical Medicine, UK [d] Makerere University, College of Humanities and Social Science, Uganda, [e] Palm Tree Academy Uganda, Uganda, [f] Uganda Virus Research Institute Entebbe, Uganda [g] London School of Hygiene & Tropical Medicine, UK [h] Department of Maternal, Newborn, Child and Adolescent Health, World Health Organisation, Geneva, Switzerland.

*Corresponding author: Helen A. Weiss. Present address: London School of Hygiene and Tropical Medicine, Keppel Street, London WC1E 7HT, United Kingdom *Email:* *helen.weiss@lshtm.ac.uk,* Phone number: +44 207 927 2087

For peer review only

Abstract

Objectives: Achieving good menstrual health and hygiene (MHH) is a public health challenge and there is little evidence to inform interventions. The aim of this study was to pilot-test an intervention to improve MHH and school attendance in Uganda, in preparation for a future cluster-randomised trial.

Design: Longitudinal study with pre-post evaluation of a pilot intervention

Setting: Two secondary schools in Entebbe, Uganda

Participants: Of the 473 eligible students in Secondary 2 (S2) at baseline, 450 (95.1%; 232 girls and 218 boys) consented/assented. 369 students (188 girls; 81.0%; and 181 boys; 83.0%) participated in the endline survey.

Intervention: The intervention comprised training teachers to improve delivery of government guidelines for puberty education, training in use of a menstrual kit and pain management, a drama skit, provision of analgesics and improvements to school Water and Sanitation Hygiene (WASH) facilities.

Primary and secondary outcome measures: Feasibility and acceptability of delivering the intervention. Baseline and endline quantitative surveys were conducted, with qualitative interviews conducted at endline. School attendance was assessed using self-completed daily diaries among a nested cohort of 100 female students.

Results: There were high levels of uptake of the individual and behavioural intervention components (puberty education, drama skit, MHM kit, and pain management). The proportion of girls reporting anxiety about next period decreased from 58.6% to 34.4%, and reported use of effective pain management increased from 76.4% to 91.4%. Most girls (81.4%) reported improved school toilet facilities, which improved their comfort managing menstruation. The diary data and qualitative data indicated a potential intervention impact on improving menstrual-related school absenteeism.

For peer review only - http://bmjopen.bmj.com/site/about/guidelines.xhtml

Conclusions: The pilot study showed that the multi-component MHM intervention was acceptable and feasible to deliver, and potentially effective in improving menstruation knowledge and management. A cluster-randomised trial is needed to evaluate rigorously the intervention effects on MHM and school attendance.

Strengths and limitations of the study:

- The study evaluated a multi-component menstrual health intervention that addresses individual, behavioural and environmental barriers to good menstrual health and school attendance.
- The study population are students in peri-urban secondary schools, which is important given the recognised importance of girls' secondary education to future development, and evidence that school absenteeism due to menstruation is problematic for secondary school girls.
- This is the first menstrual health intervention to address menstrual pain – a major contributor to school absence in girls
- The intervention includes boys as well as girls, as sustainable changes in menstrual management depend on addressing stigma about menstruation.
- The conclusions are limited due to the lack of a control group, which means that the improvements seen in knowledge, school attendance and wellbeing may reflect the girls being older at endline or differences in attendance in different terms.

For peer review only - http://bmjopen.bmj.com/site/about/guidelines.xhtml

INTRODUCTION

Many girls and women globally lack the knowledge, materials and facilities for safely managing menstruation without stigma.(1-4) Adequate menstrual hygiene management (MHM) is defined as i) using clean menstrual management material to absorb or collect menstrual blood, ii) that can be changed in privacy as often as necessary for the duration of a menstrual period, iii) using soap and water for washing the body as required, iv) having access to safe and convenient facilities to dispose of used menstrual management materials, v) understanding the basic facts linked to the menstrual cycle and vi) how to manage it with dignity and without discomfort or fear.(5) A recent systematic review of menstrual experiences among women and adolescent girls globally highlighted that interventions must address a broad range of issues including addressing stigma, knowledge, social support, restrictive behavioural expectations, and the physical and economic environment.(6) Aligned with this, the term menstrual health and hygiene (MHH) encompasses both MHM and the broader systemic factors that link menstruation with health, well-being, gender equality, education, equity, empowerment, and rights.(5)

Effective MHH interventions may lead to sustained benefits for health, productivity (3, 4, 7) and the environment (8) but there has been little rigorous evaluation of interventions to guide policies.(9-11) Schools provide an important setting for addressing MHH challenges concerning stigma, lack of menstrual literacy and goods (menstrual products, improved water, sanitation and hygiene (WASH) facilities and pain management).(12) In 2014, the “MHM in Ten” initiative developed an agenda for addressing the barriers facing girls in schools in low-income contexts.(13) The first priority of the initiative is to generate rigorous evidence on whether improving MHM improves school attendance.(9, 10, 14) A systematic review published in 2016 (15) concluded there was promising evidence of the effectiveness of MHH interventions on educational outcomes, but insufficient evidence of effect due to a small number of trials (N=8), a high risk of bias, and substantial heterogeneity. An earlier systematic review (7) of intervention and observational studies found that of the 11 studies (only one

For peer review only

RCT) investigating the association between MHM and urogenital infections, seven found an increased risk associated with “worse” MHM (defined differently for each study but generally meaning not using disposable sanitary pads), one found the reverse to be true (an increased risk from using disposable sanitary pads), and three found no association.

The aim of this study was to pilot-test a multi-component school-based MHH intervention (“MENISCUS”) and to prepare for a future cluster-randomised trial (CRT) which will evaluate the impact of the intervention on secondary school attendance, performance, menstruation knowledge, health and wellbeing outcomes in Uganda. Formative work in Wakiso District, Uganda, showed that secondary school girls reported substantial embarrassment and fear of teasing related to menstruation, reporting that this, together with menstrual pain and lack of effective materials for menstrual hygiene management, led to school absenteeism, especially in schools with low socio-economic catchment populations.(16) The issue is recognised by the Ugandan Government, which has the political will to improve MHM.(17) However the Government MHM guidelines for schools are not consistently implemented (18) for reasons including lack of funding, unclear roles and responsibilities for MHM, with issues largely left to the senior teachers.

The objectives of the pilot study are to: i) Assess the feasibility and acceptability of implementing a combined package of MHH intervention elements developed in the formative work, delivered to a whole secondary school year for nine months; ii) Assess the outcomes of the intervention package at baseline and endline (knowledge and attitudes towards puberty, menstruation and pain management); iii) Pilot the use of daily diaries to estimate school attendance, and compare attendance with estimates using registers, observation visits and retrospective self-report; iv) Estimate school retention over a 9 month period.

For peer review only - http://bmjopen.bmj.com/site/about/guidelines.xhtml

METHODS

Study setting and participants

This pilot study was conducted in Entebbe Municipality in Wakiso District, Uganda. Entebbe has 13 registered secondary schools. Two day schools (one government and one private), both with students of low socio-economic status (SES), were purposively selected. In this context, low SES schools are characterised by parents with low income, lower education achievement and under resourced in terms of low grade of teachers and facilities such as infrastructure. In these schools, poor student-teacher relationship and personal hygiene were common, and are key barriers to good MHM practices and academic performance. Eligible participants were all male and female students in the second school year (Secondary 2 (S2)). Written informed consent was sought from students aged $\geq$18 years, and from the parents/caretakers of those aged <18 years, with student assent. We also received written informed consent from teachers and parents/caretakers participating in interviews. Participation was voluntary, and confidentiality was ensured by conducting interviews in a private setting and keeping the data collected on secure servers without identifying information.

Intervention package

We previously conducted a study in this setting to develop the intervention and found that an effective MHH intervention needs to address stigma, education, attitudes and psychosocial well-being, and provide the goods defined above.(16) Following this, we finalised the pilot MENISCUS intervention, informed by social cognitive theory (SCT) which postulates that learning occurs in a social context and that personal factors, behavioural patterns and environmental aspects have bidirectional influences on one another.(19) Using the core constructs of SCT, we developed a Theory of Change (ToC) (Figure 1) with stakeholders to articulate the aims of the intervention: i) increase girls' self-efficacy to manage their menstruation (e.g. through provision of an MHM kit and pain-management options); ii) use observational learning to reinforce girls' learning and that of boys, teachers and parents to

For peer review only - http://bmjopen.bmj.com/site/about/guidelines.xhtml

create a more supportive MHM environment (through drama); and iii) provide positive reinforcement for behaviour change (through improving WASH facilities and reduced leakage/pain as a result of better MHM).

The intervention was implemented in partnership with a local NGO, WoMena Uganda, and consisted of the following. Further details are given in Supplementary Table 1:

i. Puberty education: We used a Training of Trainers (ToT) model in which WoMena Uganda worked with an educational consultant and her assistant to train 30 teachers from the two schools to improve delivery of Ugandan Government guidelines for puberty education to their students. The trained teachers were expected to develop a plan for delivering puberty training in their school and deliver the specified number of sessions to both male and female students.
ii. Drama Skit: To address stigma, attitudes and psychosocial issues about menstruation, WoMena Uganda facilitated students, with support from the school drama teacher, to develop and perform drama skits. Topics included managing pain, teasing, parental responsibilities for supporting girls with menstruation and tracking the menstrual cycle.
iii. Menstrual management kit: A kit, including a pack of 4 AFRipads reusable menstrual pads, a small towel, soap, water bottle, underwear, a mirror and menstrual calendar, was distributed to all girls in S2. WoMena Uganda trained teachers and prefects to deliver education sessions on menstruation to girls and boys on MHH, pain relief strategies such as stretching, using a hot-water bottle and use of the re-usable pads to girls only.
iv. Pain management: The research team supplied each girl with vouchers to exchange for up to six paracetamol tablets per month from the school nurse, senior teacher or a local pharmacy.
v. WASH facilities: The research team improved school WASH facilities by improving access to water close to the sanitation facilities e.g. provision of water drums and stands (one with clean water and one with soapy water), installing locks, repairing broken doors, providing bins and providing toilet-paper holders.

For peer review only - http://bmjopen.bmj.com/site/about/guidelines.xhtml

Study design and evaluation

The design was a longitudinal study with pre-post evaluation of a pilot intervention. All participants were asked to self-complete a quantitative survey at baseline (October 2017) and endline (August 2018) on tablets using Open-Data-Kit software. Questions included knowledge of puberty and menstruation, perceptions of menstruation (using a 5-point Likert scale) (Table 1), menstrual management practices at last menstrual period (LMP), reported pain and pain management strategies, leakages during LMP, anxiety, and the 25-item Strengths and Difficulties Questionnaire (SDQ) which is globally used to screen for behavioural markers of potential mental health problems in children and adolescents, covering four domains (hyperactivity, conduct problems, peer problems and emotional problems).(20-22)

Table 1: Menstruation knowledge, myth and perception questions

Topic	
1. Menstruation knowledge statements (response True/False)	Adolescence is the time between puberty and adulthood
	Changes in the body during puberty happen because of hormones
	The physical changes related to puberty usually start between 10 and 14 years of age in girls, and between 12 and 16 in boys
	Menstrual blood comes from the stomach where food is digested
	Women stop menstruating after the age of about 45-50
	Menstruation in girls and women is normal
	Pregnant women menstruate
	When a girl gets her first period, her body is ready to have children
	During her period a girl can get pregnant
2.Myth statements (response True/False)	Painkillers cause problems having children
	When a girl has her period she is unclean
	Sanitary pads can cause sickness or infection
	It is healthy for a girl to run, dance or cycle during her period
3. Menstrual cycle questions (closed responses)	What is period blood?
	How long does a period usually last?
	How many days are there usually between periods?
4. Menstruation perception statements (response (Yes/No)	I prefer staying at home during my period rather than going to school
	I worry about being teased during my period
	During my period I feel less self-confident than during other days;
	During my period I avoid physical activity (e.g. walking, running)
	I feel anxious about having my next period
	Boys tease me about my period

For peer review only - http://bmjopen.bmj.com/site/about/guidelines.xhtml

	Girls tease me about my period
	I feel comfortable to talk to other girls at school about my period
	If I had a problem with managing my period, I would talk to another girl about it

A nested cohort comprised a random sub-sample of 50 girls per school assessing the feasibility of school attendance and menstruation patterns using self-completed daily diaries. Nested cohort participants were given a booklet each term for daily recording of school and class attendance, menstruation and pain.

Qualitative interviews were used to assess perceptions and acceptability of the intervention, and its perceived impact on school attendance at endline. In-depth interviews (IDIs) were conducted with 20 female students sampled purposively (menstruating S2 girls with different levels of reported baseline school attendance), 10 teachers (six females and four males) and 10 parents who had attended the drama performance. Four focus-group discussions (FGDs) were conducted (two with each sex) at the schools. Semi-structured topic guides were used for both IDIs and FGDs. Interviewers were the same sex as respondents and interviews were digitally recorded with permission. The trustworthiness of the qualitative data was assessed in accordance with the approaches suggested by Krefting (23) to ensure credibility, applicability, dependability and confirmability of the data. The training, skill and careful supervision and support of researchers in the conduct of qualitative data collection underpins this approach (24).

Research assistants conducted nine unannounced WASH assessment visits per school at baseline and during follow-up, using a standardized checklist to assess availability, accessibility and functioning in terms of sanitary waste disposal, availability of water, soap, hand-washing facilities and toilet paper; cleanliness and privacy.

Finally, a mixed-methods process evaluation was conducted to improve understanding of intervention implementation. Details of this will be reported separately.

For peer review only

Outcomes

Outcomes were knowledge of puberty and menstruation, attitudes towards menstruation and menstrual practices (assessed by the proportion of students answering all knowledge, myth and menstrual cycles correct respectively), knowledge and use of effective pain management methods (assessed by the proportion of girls knowing, and reporting using, at least one effective pain relief method during their LMP if they reported pain), psychosocial wellbeing (assessed by the mean SDQ-25 score) and school attendance (assessed by reported days missing school in the daily diaries). The planned sample size of 200 girls (and 200 boys, respectively) provides 85% power to detect an odds ratio of two between baseline and endline assuming baseline prevalence of 20%, allowing for within-individual correlation (intracluster-correlation=0.05).

Data management and analysis

Electronically-captured data were exported to Stata version 15. The paper-based diary data and WASH checklist data were entered using Microsoft Access. For the survey data, the intervention effect on binary outcomes was estimated by comparing endline versus baseline measures, using adjusted prevalence ratios (APR), adjusted prevalence difference (APD) and 95% confidence intervals (CI) estimated using marginalised standardisation from random-effects logistic regression accounting for within-individual clustering(25). This provides an estimate of the prevalence ratio and prevalence difference for outcomes assessed at endline compared with baseline, independently of potential confounding variables. Effects on continuous outcomes were similarly assessed with random-effects linear regression to estimate the adjusted mean difference (AMD) and 95%CI. All analyses were adjusted for school, gender and age (<16, $\geq$16 years) as fixed effects. Effect-moderation by school and age group were assessed using the likelihood ratio test. For the nested cohort, period-days were defined from the diary data as the days of menstruation (bleeding) plus the day prior to menstruation, with sensitivity analyses restricted to days of menstruation only. For the cohort

For peer review only - http://bmjopen.bmj.com/site/about/guidelines.xhtml

analysis, random effects logistic regression was used to estimate APR and APD, adjusting for within-girl clustering. Audio-recordings were transcribed verbatim and translated into English. Data were analysed using thematic content analysis, with two social scientists independently coding and checking results. Key themes and subthemes related to the objectives of the qualitative research were organised in a matrix and discussed by the team for appropriate interpretation.

Participant and public involvement

Community groups were involved throughout the formative work, with stakeholders' workshops in August 2016 and October 2018, and a ToC workshop in April 2017. The ToC workshop included 35 invited participants (teachers, students and parents from the MENISCUS-1 and -2 schools, representatives from the Ministry of Education and Sports (MoES), Ministry of Health (MoH), the District Education Officer, Makerere University and NGOs working on menstrual health). In October 2018, we disseminated findings from MENISCUS-2 at a stakeholders' workshop, and elicited input into the future trial design. This meeting was attended by 60 participants, including representatives from MoES, MoH, Uganda National Council for Science and Technology (UNCST), Wakiso District Local Government Makerere University, AFRIpads, WoMena and Entebbe Municipal Council. MENISCUS-1 and two schools were represented by head teachers, students and parents.

RESULTS

Socio-demographic characteristics

Of the 473 eligible S2 students at baseline, 450 (95.1%; 232 girls and 218 boys) consented/assented. Overall, 369 students (188 girls; 81.0%; and 181 boys; 83.0%) participated in the endline survey. At baseline, the mean age was 15.4 years (SD 1.31; range

For peer review only - http://bmjopen.bmj.com/site/about/guidelines.xhtml

For peer review only

12-20) for girls and 16.2 (SD=1.5; range 13-21) for boys. Follow-up ranged from 8.6 to 9.6 months (median 9.00 months). Among the girls, 222 (95.7%) and 183 (97.3%) had started menstruating at baseline and endline respectively. The majority (n=361; 80.2%) were Christian and 198 (44.0%) were of Ganda ethnicity. About half (50.6%) of participants' mothers had post-primary education, as did 73.5% of participants' fathers (excluding 74 mothers and 88 fathers where this information was unknown). Relatively few participants (n=59; 13.1%) had running water at home, and 54 (12.0%) had a flush toilet in the home.

Feasibility and acceptability of implementing the intervention

There were high levels of uptake of the individual and behavioural intervention components (puberty education, drama skit, MHM kit, and pain management). Most students (81.3%) reported attending puberty education sessions and 93.4% of girls reported receiving the MHM kit. Both schools performed the drama skit, and the qualitative interviews indicated that the skit increased MHM awareness and enabled some of the girls to talk about MHM with their parents, especially their fathers.

"When we all saw the skit, it gave us a starting point to initiate a discussion with our children. Even men got it" **(***Female Parent).*

"Trainings improved our self-esteem and confidence because nowadays we are not scared of coming to school. We are comfortable coming to school during our menstruation. Before the training some of us were shy and we couldn't stand and talk in-front of people or parents about menstruation and puberty but now we can," (FGD girls, Private school).

The WASH component was challenging to implement. At baseline, there were no sanitary disposal bins, lockable toilet paper holders, or functional water drums at either school (Table 2). Both schools received these items as part of the intervention. At endline, in the private school, all the girls' cubicles were functional, clean and with a lockable door versus only 33%

For peer review only - http://bmjopen.bmj.com/site/about/guidelines.xhtml

in the Government school, where facilities were shared with the community. Despite this, most girls (81.4%) reported improvements in toilet facilities at endline, which improved their comfort managing menstruation.

"The school environment is now conducive … during our menstruation because the water is available, they give us water mixed with liquid soap; the toilet doors have locks, there is privacy, a person cannot interrupt you while changing the pad. Toilet paper is available whenever needed and the toilets are always clean." (Female student, Private school).

Table 2: Summary of WASH facilities at baseline and during implementation

Component	Baseline (1 visit)	Follow up (8 visits)			
	Boys and girls	Girls		Boys	
	Both schools	*Private school*	*Government school*	*Private school*	*Government school*
**Bin**	0%	6/8 (75%)	8/8 (100%)	n/a	n/a
**Toilet paper**	0%	2/8 (25%)	0/8 (0%)	1/8 (13%)	1/8 (13%)
**Functioning water drum**	0%	7/8 (88%)	7/8 (88%)	7/8 (88%)	7/8 (88%)
**Functioning water & soap drum**	0%	6/8 (75%)	2/8 (25%)	5/8 (63%)	3/8 (38%)

% of the 8 visits when the WASH component was present outside at least one toilet block in the school

Knowledge, misconceptions and perceptions of puberty and menstruation

Knowledge of puberty and menstruation was poor at baseline, especially among boys. The proportion answering all nine knowledge questions correctly increased from 11.6% to 23.9% (APR=2.18, 95%CI 1.47-3.22; p<0.001) in girls, and from 4.1% to 12.7% (APR=3.07, 95%CI 1.49-6.32; p<0.001) in boys (Table 3). There was also evidence of improvements in knowledge of the menstrual cycle, particularly among girls, although endline knowledge remained poor (29.3% of girls and 7.7% of boys answering all 3 questions correctly; Table 3).

The proportion of girls answering at least eight of nine questions on their perceptions about menstruation positively (i.e. questions about self-confidence, teasing and anxiety) increased

For peer review only - http://bmjopen.bmj.com/site/about/guidelines.xhtml

from 12.2% at baseline to 27.9% at endline (APR=2.40, 95%CI: 1.59-3.62, $p<0.001$). The largest effects were reductions in the proportion of girls reporting teasing by boys about menstruation (14.4% to 8.7%, APR=0.57, 95%CI 0.34-0.97, $p=0.04$), girls feeling anxious about the next period (58.6% to 34.4%, APR=0.57, 95%CI 0.46-0.69, $p<0.001$) and girls avoiding physical activity during menstruation (47.8% to 25.7%; APR=0.55, 95%CI 0.42-0.71, $p<0.001$). There was no evidence that the intervention effect differed by age (Table 4; $p\geq0.2$ for intervention effect modification for each variable) or school ($p\geq0.1$ for effect modification for each variable; results not shown).

For peer review only

For peer review only - http://bmjopen.bmj.com/site/about/guidelines.xhtml

Table 3: Knowledge of puberty and menstruation, attitudes to menstruation and menstrual practices at baseline and endline, by gender

	Girls					
	**Baseline (n=232)**	**Endline (n=188)**	**APR (95% CI)<br>APD (95%CI)**	**Baseline (n=218)**	**Endline (n=181)**	**APR (95% CI)<br>APD (95%CI)**
All 9 knowledge questions correct[1]	27 (11.6%)	45 (23.9%)	2.18 (1.47-3.22)<br>13.4% (6.7-20.2%)<br>P<0.001	9 (4.1%)	23 (12.7%)	3.07 (1.49-6.32)<br>8.5% (3.1-13.8%)<br>P=0.002
8 knowledge questions (excluding fertility question) correct[1]	70 (30.2%)	120 (63.8%)	2.13 (1.73-2.63)<br>34.1% (25.7-42.0%)<br>P<0.001	42 (19.3%)	73 (40.3%)	1.91 (1.44-2.54)<br>18.3% (10.7-26.0%)<br>P<0.001
All 4 myth questions correct[2]	39 (16.8%)	77 (41.0%)	2.54 (1.86-3.48)<br>25.3% (17.3-33.3%)<br>P<0.001	22 (10.1%)	44 (24.3%)	2.56 (1.64-4.01)<br>15.2% (8.1-22.2%)<br>P<0.001
All 3 menstrual cycle questions[3]	12 (5.2%)	55 (29.3%)	6.38 (3.59-11.34)<br>25.8% (18.8-32.8%)<br>P<0.001	4 (1.8%)	14 (7.7%)	3.70 (1.23-11.11)<br>5.3% (1.2-9.4%)<br>P=0.02

[1] Knowledge statements were: i) Adolescence is the time between puberty and adulthood; ii) Changes in the body during puberty happen because of hormones; iii) The physical changes related to puberty usually start between 10 and 14 years of age in girls, and between 12 and 16 in boys; iv) Menstrual blood comes from the stomach where food is digested; v) Women stop menstruating after the age of about 45-50; vi) Menstruation in girls and women is normal; vii) Pregnant women menstruate; viii) When a girl gets her first period, her body is ready to have children; ix) During her period a girl can get pregnant
[2] Myth statements were: i) Painkillers cause problems having children; ii) When a girl has her period she is unclean; iii) Sanitary pads can cause sickness or infection; iv) It is healthy for a girl to run, dance or cycle during her period
[3] Menstrual cycle questions were: i) What is period blood?; ii) How long does a period usually last?; iii) How many days are there usually between periods?

For peer review only

In qualitative interviews, girls and teachers said that they thought that fear, myths and negative perceptions about menstruation were due to lack of knowledge. Participants suggested that the intervention improved knowledge about puberty, menstruation, and girls' accounts suggested a reduction of teasing by boys at school:

"Before MENISCUS, boys used to laugh at girls, for example when a girl stood up in class with her dress stained, boys would laugh at her but after MENISCUS training, they stopped laughing at girls and they now care about us" (Female student, Private school).

Management of menstruation

The proportion of girls reporting using manufactured menstrual materials exclusively (i.e. reusable or disposable pads, tampons or menstrual cups) during their LMP increased from 73.0% at baseline to 89.1% at endline (APR=1.23, 95%CI 1.12-1.35, p<0.001). At endline, most girls (n=155; 82.5%) reported using reusable pads during their LMP compared with 18.5% at baseline. There was weak evidence of a decrease in reported leakage of blood through their clothing during LMP (47.4% at baseline vs 35.3% at endline; APR=0.76, 95%CI 0.57-1.02, p=0.06), but no evidence of a difference in the proportion of girls reporting staining their underwear during their LMP (23.4% at baseline to 27.3% at endline (APR=1.19, 95%CI 0.87-1.63, p=0.29). There was no evidence of intervention effect-moderation by age or school (Table 4).

Knowledge and use of effective pain management methods

Most girls reported pain during menstruation at both baseline and endline (74.3% and 71.0%, respectively). Amongst these girls, there was evidence of an increase in the proportion who reported using painkillers during their LMP (46.7% at baseline to 60.8% at endline; APR=1.26, 95%CI 1.03-1.55, p=0.03). Results were similar by age (Table 4) and school (results not shown). There was evidence of an increase in the proportion of girls who reported using $\geq$1 effective pain relief method during their LMP (76.4% at baseline to 91.5% at endline;

For peer review only - http://bmjopen.bmj.com/site/about/guidelines.xhtml

APR=1.19, 95%CI 1.08-1.32, p=0.001). Of the 232 girls who received a voucher for painkillers, 58 (25.0%) had redeemed 78 vouchers by endline: 77% used one voucher, 16% used two, and 7% used ≥2.

The qualitative findings confirmed that non-pharmacological methods of pain relief were popular and effective.

"They [MENISCUS team] taught us how to do exercises to relieve pain and it worked for me so, the last time, I didn't use painkillers; I managed my periods by doing exercises and using reusable pads MENISCUS had provided." (Female student, Government school).

Behavioural markers of psychosocial well-being

The mean SDQ-25 score in girls decreased from 10.3 to 9.2 (p=0.006, adjusted for age and school), indicating improved behaviour and conduct. No decrease was seen among boys, in line with the ToC (9.88 at baseline vs 9.91 at endline; p=0.98).

Menstruation and school absence

In the cross-sectional surveys, a similar proportion of girls reported missing at least one school day due to menstruation in Term 2, 2017 (pre-baseline) and Term 2, 2018 (endline) (32.0% vs 32.8%; APR=0.99, 95%CI 0.76-1.28, p=0.92). Among the 100 cohort participants, data were collected from 81 students at endline. The APR associated with missing school on period-days compared to non-period days decreased from 1.84 (95%CI 1.46-2.21) at baseline to 1.16 (95%CI 0.97-1.38) at endline (p-value for moderation=0.01; Table 5). Results were similar for class attendance (Table 5). The direct observation checks found high correlation with the self-completed diaries. Girls were seen on 328/330 (99.4%) of days when their diary stated they were present and were not seen on all 37 days when the diary stated that they were absent. School registers were incomplete and rarely completed by teachers.

For peer review only - http://bmjopen.bmj.com/site/about/guidelines.xhtml

Table 4: Reported perceptions of menstruation, menstrual management, pain and pain management among girls, by age at baseline

	Age <16 years			Age > 16 years		
	**Baseline (n=115)**	**Endline (n=51)**	**APR (95% CI) APD (95% CI)**	**Baseline (n=107)**	**Endline (n=132)**	**APR (95% CI) APD (95% CI)**
>8 of 9 positive perceptions of menstruation[1]	14 (12.2%)	16 (31.4%)	2.76 (1.10-25.19)<br>21.0% (7.2-34.9%)<br>P=0.001	13 (12.2%)	35 (26.5%)	2.23 (1.26-3.95)<br>14.6% (5.2-24.1%)<br>P<0.001
Used manufactured menstrual materials only at LMP	86 (74.8%)	46 (90.2%)	1.21 (1.06-1.39)<br>15.7% (4.6-26.8%)<br>P=0.006	76 (71.0%)	117 (88.6%)	1.25 (1.10-1.42)<br>17.6% (8.0-27.2%)<br>P<0.001
Leaked blood at LMP	37 (50.0%)	12 (42.9%)	0.84 (0.54-1.30)<br>-8.2% (-27.5-11.1%)<br>P=0.41	37 (45.1%)	24 (32.4%)	0.73 (0.49-1.09)<br>-12.1% (-27.1-2.8%)<br>P=0.11
Underwear stained at LMP	31 (27.0%)	16 (31.4%)	1.07 (0.67-1.71)<br>18.4% (-11.5-15.1%)<br>P=0.79	21 (19.6%)	34 (25.8%)	1.28 (0.82-1.99)<br>5.6% (-4.3%-15.5%)<br>P=0.27
Knew >4 effective pain management methods	30 (24.2%)	37 (67.3%)	2.72 (1.93-3.84)<br>41.8% (27.9-55.7%)<br>P<0.001	30 (27.8%)	97 (72.9%)	2.60 (1.93-3.51)<br>44.5% (34.3-54.7%)<br>P<0.001
Reported pain at last period	79 (68.7%)	36 (70.6%)	1.00 (0.82-1.22)<br>-0.01% (13.6-13.4%)	86 (80.4%)	94 (71.2%)	0.89 (0.78-1.01)<br>-9.1% (-19.1-0.9%)

[1] Perception questions were i) I prefer staying at home during my period rather than going to school; ii) I worry about being teased during my period; iii) During my period I feel less self-confident than during other days; iv) During my period I avoid physical activity (e.g. walking, running); v) I feel anxious about having my next period; vi) Boys tease me about my period vii) Girls tease me about my period; viii) I feel comfortable to talk to other girls at school about my period; ix) If I had a problem with managing my period, I would talk to another girl about it

For peer review only - http://bmjopen.bmj.com/site/about/guidelines.xhtml

			P=0.99			P=0.08
Used ≥1 effective pain management method[1]	57 (72.2%)	34 (94.4%)	1.31 (1.12-1.54)<br>22.5% (10.2-34.9%)<br>P<0.001	69 (80.2%)	85 (90.4%)	1.13 (1.00-1.27)<br>10.0% (2.9-19.9%)<br>P=0.05
Used painkillers at LMP[2]	32 (40.5%)	21 (58.3%)	1.45 (0.99-2.12)<br>18.2% (-1.6%-37.5%)<br>P=0.06	45 (52.3%)	58 (61.7%)	1.16 (0.92-1.46)<br>8.3% (-4.5%-21.1%)<br>P=0.21
Used other effective methods[2]	39 (49.4%)	29 (80.6%)	1.63 (1.24-2.15)<br>31.1% (14.0-48.1%)<br>P<0.001	43 (50.0%)	72 (76.6%)	1.55 (1.25-1.92)<br>27.3% (15.2-39.4%)<br>P<0.001
Did nothing for pain at LMP[1]	20 (25.3%)	2 (5.6%)	0.22 (0.05-0.89)<br>-19.7% (-31.9- -7.5%)<br>P<0.001	17 (19.8%)	7 (7.5%)	0.36 (0.17-0.78)<br>-12.8% (-21.8-3.7%)<br>P<0.001

[1] Effective methods (painkiller, drinking water, using hot water bottle, exercise, relaxing, foods with lots of water).

[2] Among those with pain at LMP

For peer review only

For peer review only - http://bmjopen.bmj.com/site/about/guidelines.xhtml

Table 5: School and class attendance at baseline and endline among nested cohort participants, by period-day status

	Term 3 2017 (baseline) 99 girls		Term 2 2018 (end-line) 81 girls	
	Non period-day	Period-day[1]	Non period-day	Period-day
Number of days	2625	554	4111	838
N (%) not attending full day of school	8.5%	14.6%	12.7%	14.8%
APR (95% CI)[2]	1.84 (1.46-2.31)		1.16 (0.97-1.38)	
APD (95% CI)[2]	7.1% (3.1-10.2%) P<0.001		2.1% (-0.5% - 4.6%) P=0.10	
N (%) not attending all classes	11.4%	19.9%	18.2%	20.6%
APR (95% CI)[2]	1.79 (1.47-2.17)		1.15 (0.99-1.32)	
APD (95% CI)[2]	8.9% (5.4-12.5%) P<0.001		2.7% (-0.3 - 5.6%) P=0.07	

The qualitative interviews suggested that girls were more likely to attend school during menstruation at endline than at baseline, with reasons including the training on pain management, tracking their menstrual cycle, and having re-usable pads.

"Some girls totally don't come to school every menstruation period. However, in our class, missing school is now rare because we have learnt how to manage the periods, pain and we have what to use. We now also know our cycle" (Girls FGD; Government school).

"Before MENISCUS study gave us diaries, I didn't know how to track my days. I used to come to school and my uniform would get soiled. Sometimes, I could ask for a pass out to go back home during my periods. […] But when MENISCUS came, they gave us diaries, I started filling them in and now I know my days; I come to school prepared." (Female, Private school).

[1] Includes days of menses plus day prior to menses (results similar when restricted to days of menses) obtained from daily diaries for 9 months
[2] Obtained from random effects logistic regression adjusting for age and school, allowing for within-girl clustering

For peer review only - http://bmjopen.bmj.com/site/about/guidelines.xhtml

DISCUSSION

To our knowledge, this is the first MHM intervention to be pilot tested that focused equally on health promotion (psychosocial issues, stigma and knowledge) and goods (provision of pads and analgesics, and improved WASH facilities).(26)

Feasibility and accessibility of implementing the intervention

The qualitative and quantitative data showed that the intervention was feasible and acceptable to schools and stakeholders. A detailed process evaluation will be published separately. The main challenges with implementation was maintenance of the WASH component and that we did not obtain ethical approval to offer girls a menstrual cup, due to concerns about whether cup insertion could lead to damage of the hymen and affect a girl's virginity.

Potential impact of an MHH intervention on knowledge and attitudes to puberty and menstruation

The study highlighted poor baseline knowledge of menstruation, possibly due to cultural taboos and norms (16, 27), and lack of knowledge among students (28) parents and teachers hindering discussion of puberty and menstruation.(16, 29-31) Three intervention components addressed knowledge (puberty education, menstrual-management kit training, and drama skit). To address stigma and effect a school-wide change in attitudes, the education and drama sessions included boys. The qualitative results indicated a preference for a boys-only puberty education session. This pilot study showed that girls were less anxious about menstruation, and reported less teasing by boys after the intervention, supporting the Theory of Change, and previous qualitative work from Ghana.(32) It also worth noting that although the SDQ has proven reliability and validity in a number of studies across Europe, Asia, Australia and South America(21, 33, 34), and has been used widely in Africa,(22) there has only been one psychometric validation of the tool which found satisfactory internal consistency.(35)

For peer review only - http://bmjopen.bmj.com/site/about/guidelines.xhtml

At endline, compared with baseline, more girls reported use of only manufactured materials during their LMP, and using these correctly. The high uptake of reusable pads was expected, as these were provided free to all girls, but the continued use over follow-up, and favourable qualitative reports, shows that pads were acceptable. Similar findings on correct use have been seen in another study in Uganda where 98% of girls reported washing and using soap for their AFRIpads (26) and in India,(36) where a school-based health-education intervention led to improvements in washing clothes with soap, drying them in the sun and safe disposal.

Overall, the findings suggest a potential intervention effect on education and health outcomes, but the results need to be interpreted cautiously as the improvements reported may be partly due to i) older age at endline – there may have been improvements in knowledge and self-confidence in managing menstruation due to a cohort effect; ii) reporting biases - the outcome assessment team were involved with implementation and this may have caused social desirability bias affecting responses on attitudes or behaviours following the intervention; iii) biases due to the lack of a comparison group, and the endline survey being conducted in a different term to baseline. For example, girls reported higher levels of school attendance overall at baseline than at endline, and this may be due to the baseline being conducted during an examination term; iv) generalisability – the extent to which are findings will hold in other geographic or socio-economic settings is not clear.

Methods to estimate school attendance

We found strong evidence that the association between missing school and menstruation was smaller by the end of the intervention period. This aligns with data from qualitative interviews, in which girls attributed the improved school attendance during menstruation to improved pain management, knowledge to track menstrual cycles, and the provision of reusable pads. Previous studies show inconsistent evidence for a relationship between menstruation and school absenteeism.(15, 16, 37-44) Documenting school attendance is often challenging in

For peer review only - http://bmjopen.bmj.com/site/about/guidelines.xhtml

For peer review only

LMICs due to incomplete or inaccurate school registers.(7, 16) Our diaries, administered by the research team, were popular and correlated very well with direct observation.

Key recommendations

1) Multi-component focus. Past and ongoing studies tend to focus on single-component interventions.(7, 40) This study indicates synergies between the intervention elements e.g. the drama skit reinforces puberty knowledge, reduces stigma and engages parents and boys. An emerging view, aligned with our findings, is that poor MHH is a social problem and to be effective, MHH interventions in many settings should address the broader issues of menstrual stigma and literacy as well as the provision of menstrual products or improving WASH facilities.(9) A key finding from this study is that the multi-component approach addresses both individual, behavioural and environmental barriers to good MHH and school attendance. Based on our findings, these holistic MHH interventions may benefit from establishing an MHM Leadership Group (to include teachers, parents and students) responsible for ensuring each intervention element is delivered, maintained and sustained by the schools. We recommend that future MHH interventions are multi-component, which is also in line with a recent systematic review of girls' and women's experiences of menstruation in LMICs. (6) Given the limitations of this study mentioned above, a full-scale cluster randomised trial is needed to rigorously evaluate the impact of such an intervention on girls' health and wellbeing.

2) Inclusion of pain management strategies. Menstrual pain is a major contributor to school absence in girls in many settings.(16, 27, 45, 46) This is the first MHH intervention to address this issue and we found substantial improvements in pain management (using both analgesic and non-analgesic methods).(16) Further research is needed on this topic as misconceptions about use of painkillers are common in Uganda (16) and elsewhere.(47, 48)

For peer review only - http://bmjopen.bmj.com/site/about/guidelines.xhtml

3) Inclusion of boys. We recommend that MHH interventions are inclusive of boys and men. Most MHH interventions focus only on girls, yet sustainable changes in MHM depend on addressing stigma about menstruation. This study highlighted the importance of including boys which is aligned with SCT (i.e. the intervention provides positive reinforcement for behavioural change by improving the school environment). The focus on boys as well as girls is supported by WHO framework for Health Promoting Schools (49) and is aligned with a report commissioned by the Gates Foundation on Menstrual Health and Gender Equity.(50)

4) Implementation in diverse settings: Most intervention studies to date have been in primary schools, and in rural areas.(26, 45) MHH and school attendance is an issue in the peri-urban area in Entebbe where most girls can afford to buy disposable pads for some of the time.(16) A focus on MHH in secondary schools in different settings is important given the recognised importance of girls' secondary education to future development,(51) and evidence that school absenteeism due to menstruation is problematic in secondary school girls.(16, 52)

5) Piloting and validation of methods to assess the association between menstruation and school attendance. In this study, daily diaries were the optimal method to assess the association of school attendance and menstruation. This may also be the case in other settings where school registers are not reliable, although they place some burden on the girls, and may not be accurately filled if there is little trust with the people collecting the data. Diaries have the advantage of providing data on menstruation for each girl, to enable the link with school attendance, and in this study, girls used the diaries to track their periods which reduced anxiety.

CONCLUSION

This pilot study showed that the MHH intervention was acceptable and feasible to implement. There were substantial reported improvements in stigma and anxiety, pain management, and some improvement in MHH knowledge of puberty and menstruation and WASH following a

For peer review only - http://bmjopen.bmj.com/site/about/guidelines.xhtml

multi-component intervention. Based on SCT, we anticipate the results to be generalizable to other similar contexts with relatively low levels of MHM knowledge, stigma about discussing menstruation, and poor school WASH facilities. A phase-III trial is warranted to evaluate the impact of the intervention on school attendance, and on health, well-being and educational outcomes for definitive results to drive forward policy changes.

REFERENCES

1. Das P, Baker KK, Dutta A, Swain T, Sahoo S, Das BS, et al. Menstrual Hygiene Practices, WASH Access and the Risk of Urogenital Infection in Women from Odisha, India. PLoS One. 2015;10(6):e0130777.
2. Sommer M, Caruso BA, Sahin M, Calderon T, Cavill S, Mahon T, et al. A Time for Global Action: Addressing Girls' Menstrual Hygiene Management Needs in Schools. PLoS Med. 2016;13(2):e1001962.
3. Sommer M, Sahin M. Overcoming the taboo: advancing the global agenda for menstrual hygiene management for schoolgirls. Am J Public Health. 2013;103(9):1556-9.
4. Sommer M, Hirsch JS, Nathanson C, Parker RG. Comfortably, Safely, and Without Shame: Defining Menstrual Hygiene Management as a Public Health Issue. Am J Public Health. 2015;105(7):1302-11.
5. UNICEF. Guidance on menstrual health and hygiene. New York: UNICEF; 2019.
6. Hennegan J, Shannon AK, Rubli J, Schwab KJ, Melendez-Torres GJ. Women's and girls' experiences of menstruation in low- and middle-income countries: A systematic review and qualitative metasynthesis. PLoS Med. 2019;16(5):e1002803.
7. Sumpter C, Torondel B. A systematic review of the health and social effects of menstrual hygiene management. PLoS One. 2013;8(4):e62004.
8. Elledge MF, Muralidharan A, Parker A, Ravndal KT, et al. Menstrual Hygiene Management and Waste Disposal in Low and Middle Income Countries-A Review of the Literature. 2018;15(11).
9. Bobel C. The Managed Body: Developing Girls and Menstrual Health in the Global South: Palgrave Macmillan; 2018.
10. Hennegan J. Menstrual Hygiene Management and Human Rights: The Case for an Evidence-Based Approach. Women's Reproductive Health. 2017;4(3):212-31.
11. Phillips-Howard PA, Caruso B, Torondel B, Zulaika G, Sahin M, Sommer M. Menstrual hygiene management among adolescent schoolgirls in low- and middle-income countries: research priorities. Glob Health Action. 2016;9:33032.
12. United Nations International Children's Emergency Fund, World Health Organisation. Drinking Water, Sanitation and Hygiene in Schools: Global baseline report 2018. New York: . 2018.
13. United Nations International Children's Emergency Fund. MHM in Ten; Advancing the MHM agenda in schools. Third annual meeting co-hosted by Columbia University and UNICEF New York. http://www.ungei.org/2016_MHM_in_Ten_Meeting_Report.pdf. 2016.
14. UNICEF. MHM in Ten: Advancing the MHM agenda in WASH in Schools. New York2014.
15. Hennegan J, Montgomery P. Do Menstrual Hygiene Management Interventions Improve Education and Psychosocial Outcomes for Women and Girls in Low and Middle Income Countries? A Systematic Review. PLoS One. 2016;11(2):e0146985.

For peer review only - http://bmjopen.bmj.com/site/about/guidelines.xhtml

16. Miiro G, Rutakumwa R, Nakiyingi-Miiro J, Nakuya K, Musoke S, Namakula J, et al. Menstrual health and school absenteeism among adolescent girls in Uganda (MENISCUS): a feasibility study. BMC Womens Health. 2018;18(1):4.
17. MoEST. Menstruation: Breaking the silence, taking action. Kampala; 2015.
18. Ali CS. Provision of menstrual hygiene management facilities for girls and female teachers in primary and secondary schools in Uganda : study on implementation of the Ministry of Education and Sports circular. Kampala, Uganda: IRC Wash; 2018. Contract No.: 84572.
19. Bandura A. Health promotion by social cognitive means. Health Educ Behav. 2004;31(2):143-64.
20. Deighton J, Croudace T, Fonagy P, Brown J, Patalay P, Wolpert M. Measuring mental health and wellbeing outcomes for children and adolescents to inform practice and policy: a review of child self-report measures. Child Adolesc Psychiatry Ment Health. 2014;8:14.
21. Vostanis P. Strengths and Difficulties Questionnaire: research and clinical applications. Curr Opin Psychiatry. 2006;19(4):367-72.
22. Hoosen N, Davids EL, de Vries PJ, Shung-King M. The Strengths and Difficulties Questionnaire (SDQ) in Africa: a scoping review of its application and validation. Child Adolesc Psychiatry Ment Health. 2018;12:6.
23. Krefting L. Rigor in qualitative research: the assessment of trustworthiness. Am J Occup Ther. 1991;45(3):214-22.
24. Rutakumwa R, Mugisha JO, Bernays S, Kabunga E, Tumwekwase G, Mbonye M, et al. Conducting in-depth interviews with and without voice recorders: a comparative analysis. Qualitative Research. 2019:1-17.
25. Localio AR, Margolis DJ, Berlin JA. Relative risks and confidence intervals were easily computed indirectly from multivariable logistic regression. J Clin Epidemiol. 2007;60(9):874-82.
26. Hennegan J, Dolan C, Wu M, Scott L, Montgomery P. Schoolgirls' experience and appraisal of menstrual absorbents in rural Uganda: a cross-sectional evaluation of reusable sanitary pads. Reprod Health. 2016;13(1):143.
27. UNICEF Western and Central A firica Regional Office. Menstrual Hygiene in Schools in 2 countries of Francophone West Africa; Burkina Faso and Niger Case Studies. 2013.
28. Shanbhag D, Shilpa R, D'Souza N, Josphine P, Singh J, Goud BR. Perceptions regarding menstruation and Practices during menstrual cycles among high school going adolescent girls in resource limited settings around Bangalore City, Karnataka, India. . Int J Collab Res Inter Med Public Health. 2012;4(7):1353–62.
29. Chandra-Mouli V, Patel SV. Mapping the knowledge and understanding of menarche, menstrual hygiene and menstrual health among adolescent girls in low- and middle-income countries. Reprod Health. 2017;14(1):30.
30. McMahon SA, Winch PJ, Caruso BA, Obure AF, Ogutu EA, Ochari IA, et al. 'The girl with her period is the one to hang her head' Reflections on menstrual management among schoolgirls in rural Kenya. BMC Int Health Hum Rights. 2011;11:7.
31. Bosch AM, Hutter I, van Ginneken JK. Perceptions of adolescents and their mothers on reproductive and sexual development in Matlab, Bangladesh. Int J Adolesc Med Health. 2008;20(3):329-42.
32. Dolan CS, Ryus CR, Dopson S, Montgomery P, Scott L. A blind spot in girls' education: menarche and its webs of exclusion in Ghana. Journal of International Development. 2013;26(5):643±57.
33. Woerner W, Fleitlich-Bilyk B, Martinussen R, Fletcher J, Cucchiaro G, Dalgalarrondo P, et al. The Strengths and Difficulties Questionnaire overseas: evaluations and applications of the SDQ beyond Europe. Eur Child Adolesc Psychiatry. 2004;13 Suppl 2:II47-54.
34. Goodman R. The Strengths and Difficulties Questionnaire: a research note. J Child Psychol Psychiatry. 1997;38(5):581-6.

For peer review only - http://bmjopen.bmj.com/site/about/guidelines.xhtml

35. Kashala E, Elgen I, Sommerfelt K, Tylleskar T. Teacher ratings of mental health among school children in Kinshasa, Democratic Republic of Congo. Eur Child Adolesc Psychiatry. 2005;14(4):208-15.
36. Nemade D, Anjenaya S, Gujar R. Impact of health education on knowledge and practices about menstruation among adolescent school girls of Kalamboli, Navi-Mumbai. . Health Popul Perspect Issues. 2009;32(4):167–75.
37. Grant MJ, Lloyd CB, Mensch BS. Menstruation and School Absenteeism: Evidence from Rural Malawi. Comp Educ Rev. 2013;57(2):260-84.
38. van Eijk AM, Sivakami M, Thakkar MB, Bauman A, Laserson KF, Coates S, et al. Menstrual hygiene management among adolescent girls in India: a systematic review and meta-analysis. BMJ Open. 2016;6(3):e010290.
39. Oster E, Thornton R. Menstruation, Sanitary Products, and School Attendance: Evidence from a Randomized Evaluation. American Economic Journal: Applied Economics. 2011;3(1):91-100.
40. Phillips-Howard PA, Nyothach E, Ter Kuile FO, Omoto J, Wang D, Zeh C, et al. Menstrual cups and sanitary pads to reduce school attrition, and sexually transmitted and reproductive tract infections: a cluster randomised controlled feasibility study in rural Western Kenya. BMJ Open. 2016;6(11):e013229.
41. Tegegne TK, Sisay MM. Menstrual hygiene management and school absenteeism among female adolescent students in Northeast Ethiopia. BMC Public Health. 2014;14:1118.
42. Montgomery P, Ryus CR, Dolan CS, Dopson S, Scott LM. Sanitary pad interventions for girls' education in Ghana: a pilot study. PLoS One. 2012;7(10):e48274.
43. Wilson E, Reeve J, Pitt A. Education. Period. Developing an acceptable and replicable menstrual hygiene intervention. Development in Practice 2014;24(1):63±80.
44. Scott L, Dopson S, Montgomery P, Dolan C, Ryus C. Impact of Providing Sanitary Pads To Poor Girls in Africa. University of Oxford. 2009.
45. Boosey R, Prestwich G, Deave T. Menstrual hygiene management amongst schoolgirls in the Rukungiri district of Uganda and the impact on their education: a cross-sectional study. Pan Afr Med J. 2014;19:253.
46. Crofts T, J. F. Menstrual hygiene in Ugandan schools: an investigation of low-cost sanitary pads. J Water Sanitation Hyg Dev. 2012;2:50–8.
47. Kontogiorgis C, Nena E, Berberoglou E, Moschoni K, et al. Estimating Consumers' Knowledge and Attitudes Towards Over-The-Counter Analgesic Medication in Greece in the Years of Financial Crisis: The Case of Paracetamol. Pain and therapy. 2016;5(1):19-28.
48. Palos GR, Mendoza TR, Cantor SB, et al. Perceptions of analgesic use and side effects: what the public values in pain management. Journal of pain and symptom management. 2004;28(5):460-73.
49. Langford R, Bonell C, Jones H, Pouliou T, Murphy S, Waters E, et al. The World Health Organization's Health Promoting Schools framework: a Cochrane systematic review and meta-analysis. BMC Public Health. 2015;15:130.
50. Geertz A, Iyer L, Kasen P, Mazzola F, Peterson K. An opportunity to address menstrual health and gender equity. Bill & Melinda Gates Foundation; May 2016.
51. Wodon QT, Montenegro CE, Nguyen H, et al. Missed Opportunities : The High Cost of Not Educating Girls (English). The Cost of Not Educating Girls Series. Washington, D.C. : World Bank Group. http://documents.worldbank.org/curated/en/775261531234655903/Missed-Opportunities-The-High-Cost-of-Not-Educating-Girls. 2018.
52. Vashisht A, Pathak R, Agarwalla R, et al. School absenteeism during menstruation amongst adolescent girls in Delhi, India. Journal of family & community medicine. 2018;25(3):163-8.

For peer review only - http://bmjopen.bmj.com/site/about/guidelines.xhtml

Figure Legend:

Figure 1: Theory of Change for the MENISCUS intervention

Statements:

Contributors: HAW, LH, BT, DAR, JS, CB, SNe, SCF and CA contributed to the overall conception and design of the study. CK, LH, SNa, RN, KN, PN, SNM undertook the data collection. CK and HAW wrote the first draft of the manuscript. All authors contributed to the interpretation of results and drafting of the manuscript. HAW and CT did the statistical analyses. All authors take responsibility for the integrity of the data and the accuracy of the data analysis. All authors read and approved the final manuscript. HAW is the guarantor.

Funding statement: This work was supported by a United Kingdom (UK) Department for International Development (DFID), the National Institute for Health Research (NIHR), UK Medical Research Council (MRC), and Wellcome Trust Joint Global Health Trials Development Grant (number MR/P020283/1). The authors would like to thank the study participants, contact teachers in the two schools, and the Ministry of Education and Sports and Ministry of Health Uganda for their support.

Competing Interests: None declared

Disclaimer: The content is solely the responsibility of the authors and does not necessarily represent the official views of the funders.

Ethical approval: Ethics approval was received from the UVRI Research Ethics Committee, the Uganda National Council for Science and Technology (UNCST) and the London School of Hygiene and Tropical Medicine.

For peer review only - http://bmjopen.bmj.com/site/about/guidelines.xhtml

Data sharing statement: Data will be made available in the LSHTM Data Compass repository on request from the corresponding author (Helen Weiss https://orcid.org/0000-0003-3547-7936) from the website https://datacompass.lshtm.ac.uk/

Patient request for publication: Not required

For peer review only

For peer review only - http://bmjopen.bmj.com/site/about/guidelines.xhtml

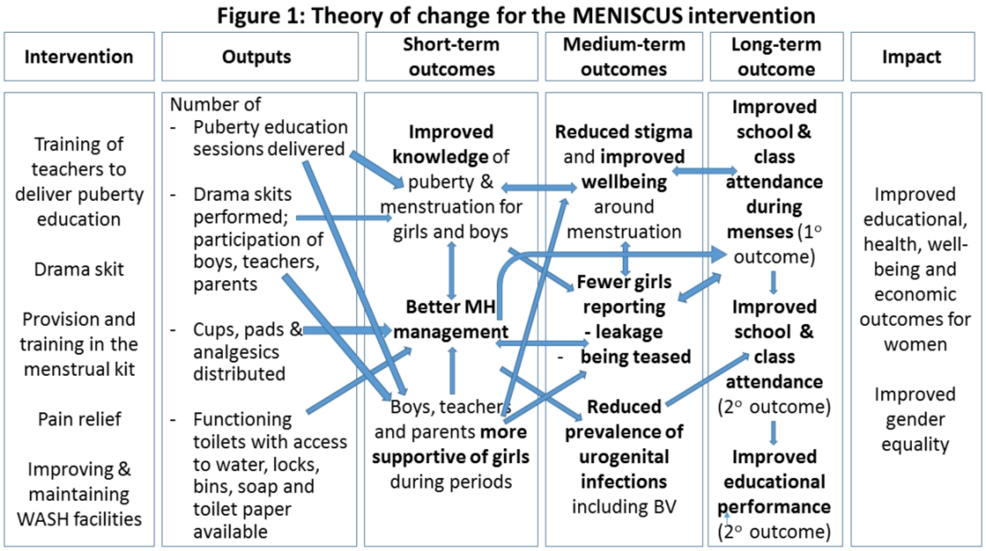

Figure 1: Theory of Change for MENISCUS intervention

338x190mm (96 x 96 DPI)

Supplementary Table 1: Description of the MENISCUS intervention

**MENISUS intervention**<br>**1. Puberty education**<br>**2. Drama skit**<br>**3. Menstrual management kit & training**<br>**4. Pain management**<br>**5. Water, Sanitation and Hygiene (WASH) improvements**
**Materials & procedures**
1. Secondary year 2 teachers from both intervention schools and other relevant teachers selected by school management received 2 days of training in how to deliver puberty education according to government guidelines using the draft National Training of Trainers Manual on MHM compiled by the Ugandan Ministry of Education and Sports (MoES).<br>2. School Drama Groups with students from Secondary year 1 to year 4 received two facilitated drama skit sessions (one on menstrual health and one on the drama skit process) followed by follow-up visits to drama group practices. The MHM drama was performed by the drama groups at a parental meeting at each school (an annual general meeting and an specifically organised parental meeting). The meetings were attended by teachers, parents of secondary school students and some secondary year 2 students.<br>3. Secondary year 2 girl and boy students were invited to participate in an MHM training session. Secondary year 2 girl students were provided with a menstrual kit consisting of a pack of AFRIpads reusable pads, a towel, soap, water bottle, knickers and menstrual calendar and an educational session on safe use and care of reusable menstrual products as well as pain management methods. Follow-up sessions were provided throughout the school year.<br>4. Students were provided with one voucher at baseline and throughout the study to redeem painkillers at school or a local pharmacy. All used vouchers were replaced per month.<br>5. WASH improvements consisted of installing locks, repairing broken doors, providing bins and toilet paper holders fixed to the wall, liquid hand washing soap and water drums.
**Who provided**
1. An independent educational consultant with support from WoMena Uganda staff members trained teachers from each school who are responsible for delivering puberty education in 2-days of puberty education training. The educational consultant was a professional trainer with expertise in education management.WoMena Uganda is a non-governmental organisation with expertise in programme design, monitoring and evaluation and education for menstrual health interventions. WoMena has a training team of young Ugandan menstrual health trainers (aged 20 -28, with educational backgrounds in social care, nursing, education), led by the Training Coordinator. The puberty education sessions were supported by the Training Coordinator and a trainer.<br>2. Two facilitated drama skit sessions were delivered by the WoMena Uganda Training Coordinator (supported by a Drama Skit facilitation guide developed by WoMena Uganda). Follow-up sessions with the Drama Groups were led by the school Drama Teacher and supported by an independent drama skit consultant, who was engaged in Drama Skit activities during MENISCUS-1 (a previous formative study carried out in Entebbe).<br>3. The MHM training session was delivered by selected schoolteachers and peers who had been trained in its delivery by WoMena Uganda. Training sessions were supported by a

For peer review only

team of six Womena Uganda trainers and the Training Coordinator. Follow-up sessions were provided by WoMena Uganda.<br>4. Painkiller vouchers could be redeemed for painkillers (paracetamol) from selected school teachers, nurse, senior women teachers and a local pharmacy.<br>5. WASH improvements were made by MRC/UVRI and maintained by the schools.
**How**
1. An education consultant and WoMena Uganda provided 2 days of group training in puberty education to teachers.<br>2. Two facilitated Drama Skit introduction sessions were held in each school by WoMena Uganda. Follow up sessions throughout terms 3 and 1 (of year 3) were carried out by the drama skit consultant. The drama performances were arranged by the school Drama Teachers in collaboration with school management as part of parental meetings.<br>3. Menstrual management kits and training of school teachers and peers was provided in group training sessions by WoMena Uganda<br>4. Painkillers and vouchers were delivered to schools / students by the MRC/UVRI and LSHTM research team..<br>5. WASH improvements were made by MRC/UVRI and LSHTM and maintained by the schools.
**Where**
Staff training in puberty education was conducted outside the schools. All other activities with students and staff were conducted in schools.
**When and how much**
1. Two days of training in puberty education to teachers delivered in April 2017.<br>2. Two, two-hour introductions facilitated by WoMena Uganda were delivered in October and November 2017. 21 follow up visits to drama skit practices were carried out between November 2017 and June 2018 (10 follow-up session planned). Two drama skit performances were carried out in July 2018.<br>3. Two, one-day Training of Trainers sessions delivered by WoMena Uganda in two schools to 11 female teachers, 11 female students and 2 males (drama teacher and school nurse) in May 2017. A one-day refresher training was held in September 2017, for selected female students (5) and female teachers (6) before delivery of training to students (not planned). Training to Secondary year 2 students (boys and girls) was delivered over 8 days in October and November (planned 7 days) over 17 training sessions (15 planned).

For peer review only

STROBE Statement—Checklist of items that should be included in reports of ***cohort studies***

	Item No	Recommendation	Page number
**Title and abstract**	1	(*a*) Indicate the study's design with a commonly used term in the title or the abstract	1
		(*b*) Provide in the abstract an informative and balanced summary of what was done and what was found	2
**Introduction**			
Background/rationale	2	Explain the scientific background and rationale for the investigation being reported	4-5
Objectives	3	State specific objectives, including any prespecified hypotheses	5
**Methods**			
Study design	4	Present key elements of study design early in the paper	8
Setting	5	Describe the setting, locations, and relevant dates, including periods of recruitment, exposure, follow-up, and data collection	8,11
Participants	6	(*a*) Give the eligibility criteria, and the sources and methods of selection of participants. Describe methods of follow-up	8,11
		(*b*) For matched studies, give matching criteria and number of exposed and unexposed	n/a
Variables	7	Clearly define all outcomes, exposures, predictors, potential confounders, and effect modifiers. Give diagnostic criteria, if applicable	6, 9
Data sources/ measurement	8*	For each variable of interest, give sources of data and details of methods of assessment (measurement). Describe comparability of assessment methods if there is more than one group	8,9
Bias	9	Describe any efforts to address potential sources of bias	10
Study size	10	Explain how the study size was arrived at	10
Quantitative variables	11	Explain how quantitative variables were handled in the analyses. If applicable, describe which groupings were chosen and why	10
Statistical methods	12	(*a*) Describe all statistical methods, including those used to control for confounding	10
		(*b*) Describe any methods used to examine subgroups and interactions	10
		(*c*) Explain how missing data were addressed	n/a
		(*d*) If applicable, explain how loss to follow-up was addressed	n/a
		(*e*) Describe any sensitivity analyses	n/a
**Results**			
Participants	13*	(a) Report numbers of individuals at each stage of study—eg numbers potentially eligible, examined for eligibility, confirmed eligible, included in the study, completing follow-up, and analysed	11
		(b) Give reasons for non-participation at each stage	n/a
		(c) Consider use of a flow diagram	n/a
Descriptive data	14*	(a) Give characteristics of study participants (eg demographic, clinical, social) and information on exposures and potential confounders	11
		(b) Indicate number of participants with missing data for each variable of interest	11
		(c) Summarise follow-up time (eg, average and total amount)	11
Outcome data	15*	Report numbers of outcome events or summary measures over time	13-17
Main results	16	(*a*) Give unadjusted estimates and, if applicable, confounder-adjusted estimates and their precision (eg, 95% confidence interval). Make clear	13-17

For peer review only - http://bmjopen.bmj.com/site/about/guidelines.xhtml

		which confounders were adjusted for and why they were included	
		(*b*) Report category boundaries when continuous variables were categorized	13-17
		(*c*) If relevant, consider translating estimates of relative risk into absolute risk for a meaningful time period	n/a
Other analyses	17	Report other analyses done—eg analyses of subgroups and interactions, and sensitivity analyses	13 and 15
**Discussion**			
Key results	18	Summarise key results with reference to study objectives	19
Limitations	19	Discuss limitations of the study, taking into account sources of potential bias or imprecision. Discuss both direction and magnitude of any potential bias	19
Interpretation	20	Give a cautious overall interpretation of results considering objectives, limitations, multiplicity of analyses, results from similar studies, and other relevant evidence	20-21
Generalisability	21	Discuss the generalisability (external validity) of the study results	21
**Other information**			
Funding	22	Give the source of funding and the role of the funders for the present study and, if applicable, for the original study on which the present article is based	25

*Give information separately for exposed and unexposed groups.

Note: An Explanation and Elaboration article discusses each checklist item and gives methodological background and published examples of transparent reporting. The STROBE checklist is best used in conjunction with this article (freely available on the Web sites of PLoS Medicine at http://www.plosmedicine.org/, Annals of Internal Medicine at http://www.annals.org/, and Epidemiology at http://www.epidem.com/). Information on the STROBE Initiative is available at http://www.strobe-statement.org.

For peer review only
